# Supplementary material for: Characterization of Indoor Molds after Ajka Red Mud Spill, Hungary
Source: Pathogens. 2023 Dec 26;13(1):22. doi: 10.3390/pathogens13010022 (PMC10820486; doi:10.3390/pathogens13010022)
Supplement: Supplementary file 1 [file pathogens-13-00022-s001.zip › pathogens-2739593-supplementary.pdf]

Characterization of indoor molds after Ajka red mud spill, Hungary

Supplementary material

Contents

Comments 1

Water activity 2

Substrate 16

pH 31

Comments

- ***Figures:***
  - **Lines** indicate the average colony diameter calculated on the basis of 3 replicates in a given treatment
  - **Dots** also represent the average for the days, added to the graphs for possible overlapping lines *(jittered dots, not the actual values, but "shaken" so they don't overlap, and more visible)*
  - The **shaded areas** around the lines represent the scatter of the colony diameter
    - often the dimensions of the 3 replicates are exactly the same, so there is no scatter
- ***ANOVA:***
  - for each strain, the effect of treatments was compared by ANOVA
  - the results obtained may not be reliable because there are few replicates and often the dimensions of the replicates are exactly the same within a treatment
  - in the ANOVA table, **p-value** (`Pr(>F)`) if less than 0.05, then at least one of the means of the treatments is different from the others
- ***Dunnett-test:***
  - pairwise comparison of individual treatments
  - in all cases the control group is the reference for comparisons
  - in the table, `diff` is the difference from the control group (`lwr.ci` & `upr.ci` are the corresponding 95% confidence intervals)
  - and `pval` is the p-value, if less than 0.05 then there is a significant difference between the control and the other treatment

Water activity

```
[1] "T398A"
```

Figure S1. Water activity − T398A

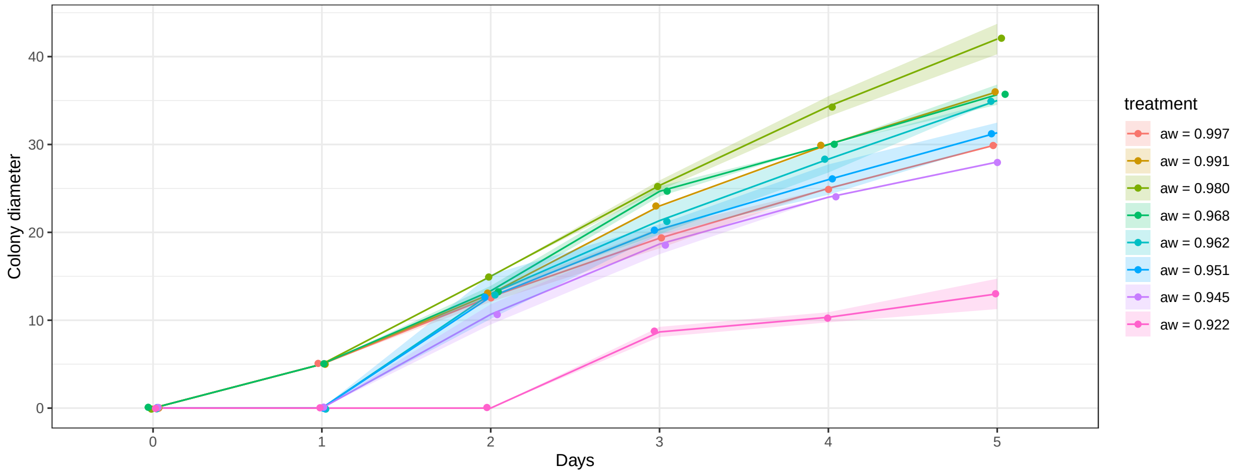


```
[1] "ANOVA results:"
                        Df Sum Sq Mean Sq F value Pr(>F)    
data_stat_id$treatment  7  15384    2198     743 <2e-16 ***
Residuals              16     47       3                   
---
Signif. codes:  0 '***' 0.001 '**' 0.01 '*' 0.05 '.' 0.1 ' ' 1
[1] ""
[1] "############"
[1] ""
[1] "Dunnett-test results:"

  Dunnett's test for comparing several treatments with a control :  
    95% family-wise confidence level

$`aw = 0.997`
                           diff    lwr.ci    upr.ci    pval    
aw = 0.991-aw = 0.997   15.0000  10.8950  19.1050 2.4e-09 ***
aw = 0.980-aw = 0.997   29.6667  25.5617  33.7717 < 2e-16 ***
aw = 0.968-aw = 0.997   16.6667  12.5617  20.7717 8.9e-09 ***
aw = 0.962-aw = 0.997    5.6667   1.5617   9.7717  0.0055 ** 
aw = 0.951-aw = 0.997   -1.6667  -5.7717   2.4383  0.7388    
aw = 0.945-aw = 0.997  -10.6667 -14.7717  -6.5617 3.1e-06 ***
aw = 0.922-aw = 0.997  -60.0000 -64.1050 -55.8950 < 2e-16 ***

---
Signif. codes:  0 '***' 0.001 '**' 0.01 '*' 0.05 '.' 0.1 ' ' 1

[1] ""
[1] "################################"
[1] "################################"
[1] ""
[1] "T398B"
```

Figure S2. Water activity − T398B

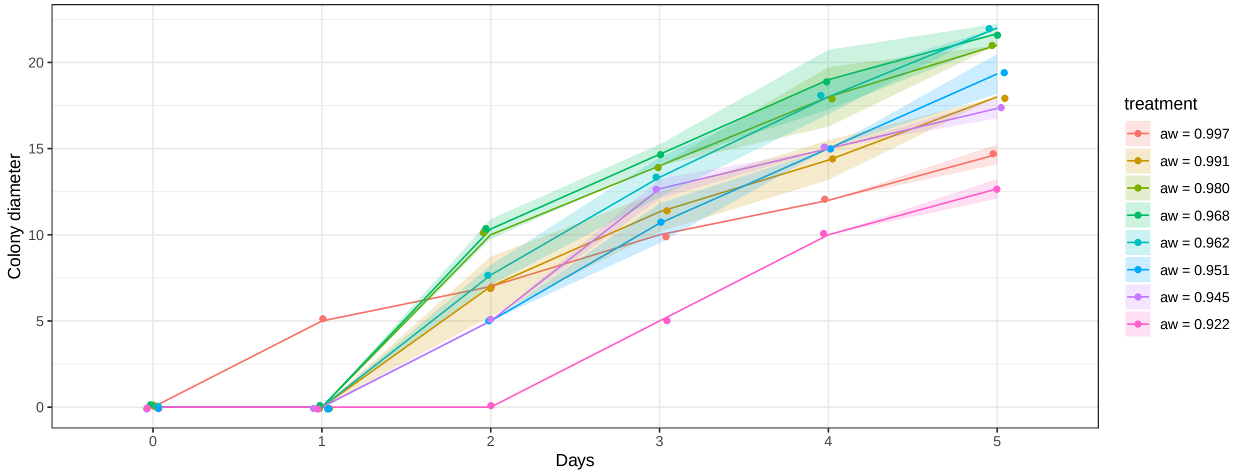


```
[1] "ANOVA results:"
                       Df Sum Sq Mean Sq F value  Pr(>F)    
data_stat_id$treatment  7   3005     429     210 1.5e-14 ***
Residuals              16     33       2                    
---
Signif. codes:  0 '***' 0.001 '**' 0.01 '*' 0.05 '.' 0.1 ' ' 1
[1] ""
[1] "############"
[1] ""
[1] "Dunnett-test results:"

  Dunnett's test for comparing several treatments with a control :  
    95% family-wise confidence level

$`aw = 0.997`
                          diff    lwr.ci    upr.ci    pval    
aw = 0.991-aw = 0.997   2.0000   -1.4102    5.4102  0.4001    
aw = 0.980-aw = 0.997  14.3333   10.9231   17.7436 9.4e-11 ***
aw = 0.968-aw = 0.997  17.0000   13.5898   20.4102 6.3e-14 ***
aw = 0.962-aw = 0.997  12.3333    8.9231   15.7436 2.3e-08 ***
aw = 0.951-aw = 0.997   1.3333   -2.0769    4.7436  0.7669    
aw = 0.945-aw = 0.997   1.3333   -2.0769    4.7436  0.7669    
aw = 0.922-aw = 0.997 -21.0000  -24.4102  -17.5898 5.3e-10 ***

---
Signif. codes:  0 '***' 0.001 '**' 0.01 '*' 0.05 '.' 0.1 ' ' 1

[1] ""
[1] "###############################"
[1] "###############################"
[1] ""
[1] "T399"
```

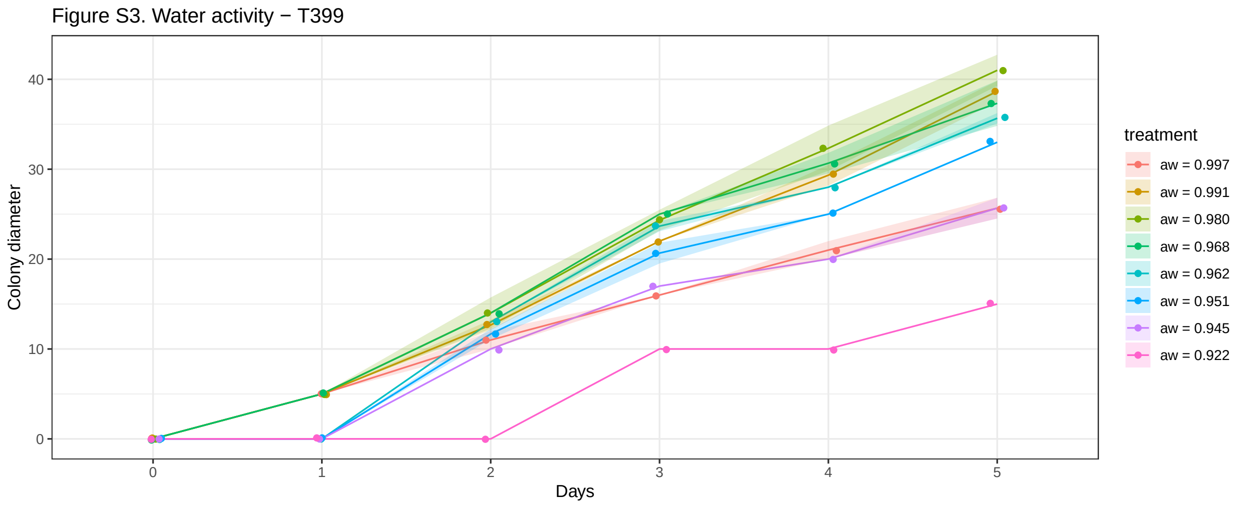


```
[1] "ANOVA results:"
                        Df Sum Sq Mean Sq F value Pr(>F)
data_stat_id$treatment  7  15187    2170     579 <2e-16 ***
Residuals              16     60       4
---
Signif. codes:  0 '***' 0.001 '**' 0.01 '*' 0.05 '.' 0.1 ' ' 1
[1] ""
[1] "############"
[1] ""
[1] "Dunnett-test results:"

  Dunnett's test for comparing several treatments with a control :
    95% family-wise confidence level

$`aw = 0.997`
                          diff    lwr.ci    upr.ci    pval
aw = 0.991-aw = 0.997  29.000  24.3783  33.6217 < 2e-16 ***
aw = 0.980-aw = 0.997  38.000  33.3783  42.6217 < 2e-16 ***
aw = 0.968-aw = 0.997  33.333  28.7116  37.9551 < 2e-16 ***
aw = 0.962-aw = 0.997  21.667  17.0449  26.2884 4.0e-12 ***
aw = 0.951-aw = 0.997  11.667   7.0449  16.2884 6.8e-06 ***
aw = 0.945-aw = 0.997  -6.000 -10.6217  -1.3783  0.0089 **
aw = 0.922-aw = 0.997 -43.667 -48.2884 -39.0449 < 2e-16 ***

---
Signif. codes:  0 '***' 0.001 '**' 0.01 '*' 0.05 '.' 0.1 ' ' 1

[1] ""
[1] "################################"
[1] "################################"
[1] ""
[1] "T401A"
```

Figure S4. Water activity − T401A

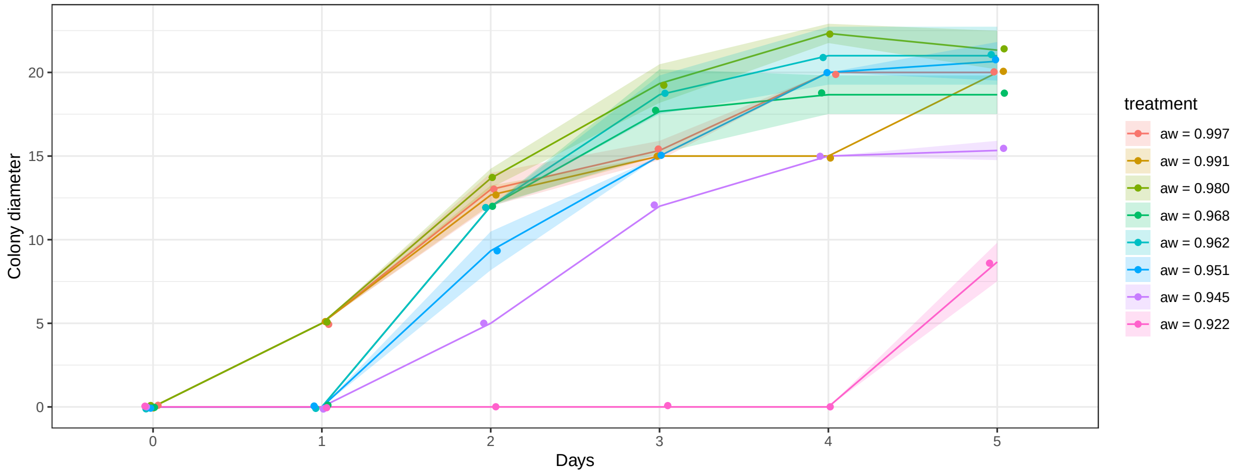


```
[1] "ANOVA results:"
                        Df Sum Sq Mean Sq F value Pr(>F)
data_stat_id$treatment  7  11204    1601     272  2e-15 ***
Residuals               16     94       6
---
Signif. codes:  0 '***' 0.001 '**' 0.01 '*' 0.05 '.' 0.1 ' ' 1
[1] ""
[1] "############"
[1] ""
[1] "Dunnett-test results:"

  Dunnett's test for comparing several treatments with a control :
    95% family-wise confidence level

$`aw = 0.997`
                            diff   lwr.ci    upr.ci    pval
aw = 0.991-aw = 0.997  -5.66667 -11.4515   0.11821  0.0561 .
aw = 0.980-aw = 0.997   8.33333   2.5485  14.11821  0.0038 **
aw = 0.968-aw = 0.997  -6.33333 -12.1182  -0.54846  0.0293 *
aw = 0.962-aw = 0.997  -0.66667  -6.4515   5.11821  0.9995
aw = 0.951-aw = 0.997  -8.33333 -14.1182  -2.54846  0.0038 **
aw = 0.945-aw = 0.997 -26.00000 -31.7849 -20.21513 2.7e-11 ***
aw = 0.922-aw = 0.997 -64.66667 -70.4515 -58.88179 < 2e-16 ***

---
Signif. codes:  0 '***' 0.001 '**' 0.01 '*' 0.05 '.' 0.1 ' ' 1

[1] ""
[1] "################################"
[1] "################################"
[1] ""
[1] "T401B"
```

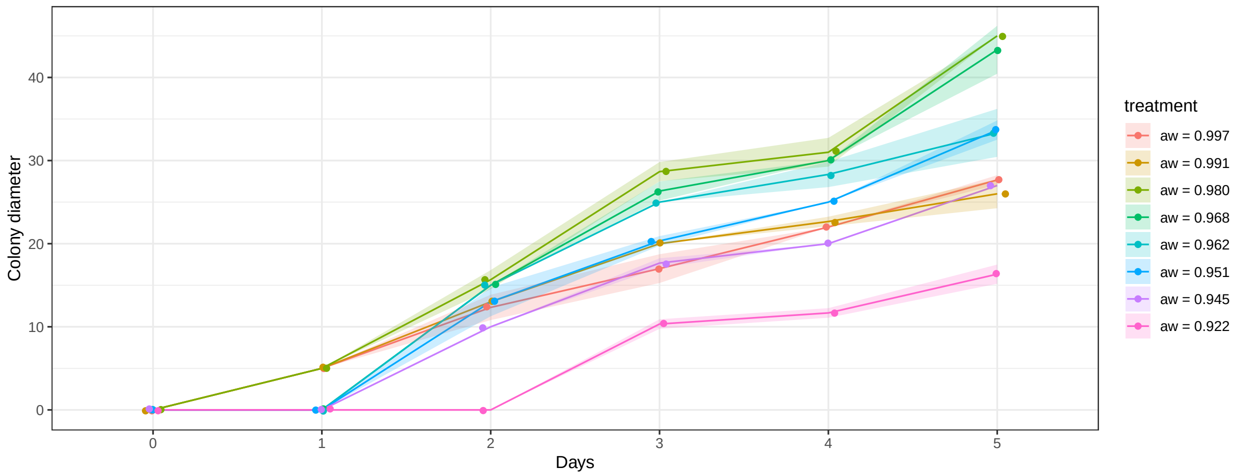


```
[1] "ANOVA results:"
                        Df Sum Sq Mean Sq F value  Pr(>F)    
data_stat_id$treatment  7  14843    2120     278 1.7e-15 ***
Residuals              16    122       8                    
---
Signif. codes:  0 '***' 0.001 '**' 0.01 '*' 0.05 '.' 0.1 ' ' 1
[1] ""
[1] "############"
[1] ""
[1] "Dunnett-test results:"

  Dunnett's test for comparing several treatments with a control :  
    95% family-wise confidence level

$`aw = 0.997`
                           diff    lwr.ci   upr.ci     pval    
aw = 0.991-aw = 0.997   2.6667  -3.9237   9.257   0.7413    
aw = 0.980-aw = 0.997  41.3333  34.7430  47.924  4.6e-15 ***
aw = 0.968-aw = 0.997  30.6667  24.0763  37.257  2.3e-10 ***
aw = 0.962-aw = 0.997  17.6667  11.0763  24.257  1.9e-06 ***
aw = 0.951-aw = 0.997   8.0000   1.4096  14.590   0.0144 *  
aw = 0.945-aw = 0.997  -9.3333 -15.9237  -2.743   0.0043 ** 
aw = 0.922-aw = 0.997 -45.6667 -52.2570 -39.076  < 2e-16 ***

---
Signif. codes:  0 '***' 0.001 '**' 0.01 '*' 0.05 '.' 0.1 ' ' 1

[1] ""
[1] "################################"
[1] "################################"
[1] ""
[1] "TD12"
```

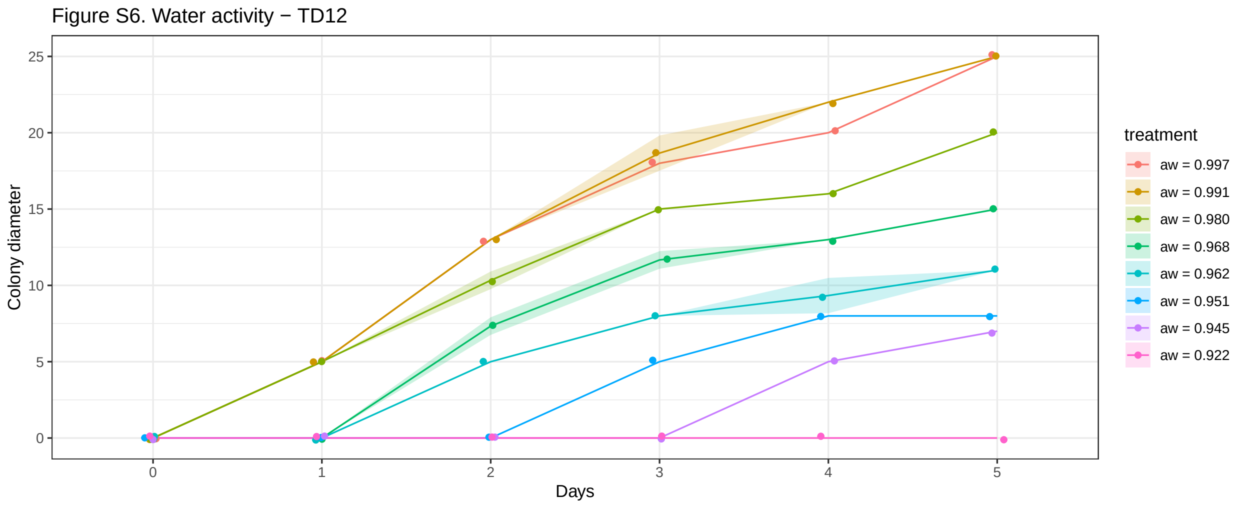


```
[1] "ANOVA results:"
                       Df Sum Sq Mean Sq F value Pr(>F)
data_stat_id$treatment  7  21137    3020    6039 <2e-16 ***
Residuals              16      8       0
---
Signif. codes:  0 '***' 0.001 '**' 0.01 '*' 0.05 '.' 0.1 ' ' 1
[1] ""
[1] "############"
[1] ""
[1] "Dunnett-test results:"

  Dunnett's test for comparing several treatments with a control :
    95% family-wise confidence level

$`aw = 0.997`
                           diff     lwr.ci    upr.ci   pval
aw = 0.991-aw = 0.997   2.6667   0.97905    4.3543 0.0016 **
aw = 0.980-aw = 0.997 -14.6667 -16.35429 -12.9790 <2e-16 ***
aw = 0.968-aw = 0.997 -34.0000 -35.68762 -32.3124 <2e-16 ***
aw = 0.962-aw = 0.997 -47.6667 -49.35429 -45.9790 <2e-16 ***
aw = 0.951-aw = 0.997 -60.0000 -61.68762 -58.3124 <2e-16 ***
aw = 0.945-aw = 0.997 -69.0000 -70.68762 -67.3124 <2e-16 ***
aw = 0.922-aw = 0.997 -81.0000 -82.68762 -79.3124 <2e-16 ***

---
Signif. codes:  0 '***' 0.001 '**' 0.01 '*' 0.05 '.' 0.1 ' ' 1

[1] ""
[1] "################################"
[1] "################################"
[1] ""
[1] "TD16"
```

Figure S7. Water activity − TD16

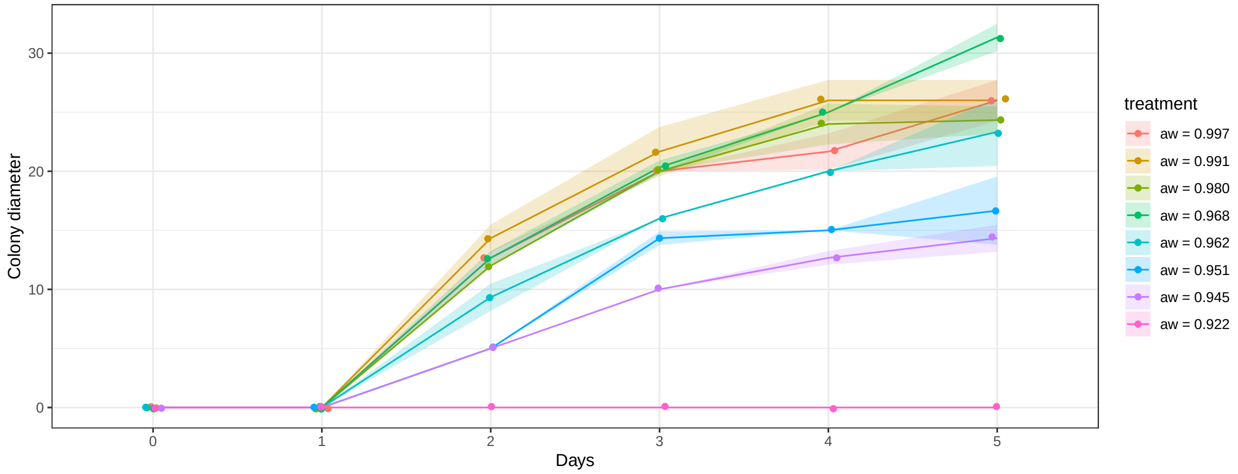


```
[1] "ANOVA results:"
                       Df Sum Sq Mean Sq F value Pr(>F)
data_stat_id$treatment  7  19509    2787     582 <2e-16 ***
Residuals              16     77       5
---
Signif. codes:  0 '***' 0.001 '**' 0.01 '*' 0.05 '.' 0.1 ' ' 1
[1] ""
[1] "############"
[1] ""
[1] "Dunnett-test results:"

  Dunnett's test for comparing several treatments with a control :
    95% family-wise confidence level

$`aw = 0.997`
                             diff    lwr.ci    upr.ci     pval
aw = 0.991-aw = 0.997   7.6667   2.4423   12.8910 0.00337 **
aw = 0.980-aw = 0.997   0.0000  -5.2244    5.2244 1.00000
aw = 0.968-aw = 0.997   9.0000   3.7756   14.2244 0.00071 ***
aw = 0.962-aw = 0.997 -11.6667 -16.8910   -6.4423 8.2e-05 ***
aw = 0.951-aw = 0.997 -29.3333 -34.5577 -24.1090 6.7e-13 ***
aw = 0.945-aw = 0.997 -38.3333 -43.5577 -33.1090 < 2e-16 ***
aw = 0.922-aw = 0.997 -80.3333 -85.5577 -75.1090 < 2e-16 ***

---
Signif. codes:  0 '***' 0.001 '**' 0.01 '*' 0.05 '.' 0.1 ' ' 1

[1] ""
[1] "################################"
[1] "################################"
[1] ""
[1] "TD22"
```

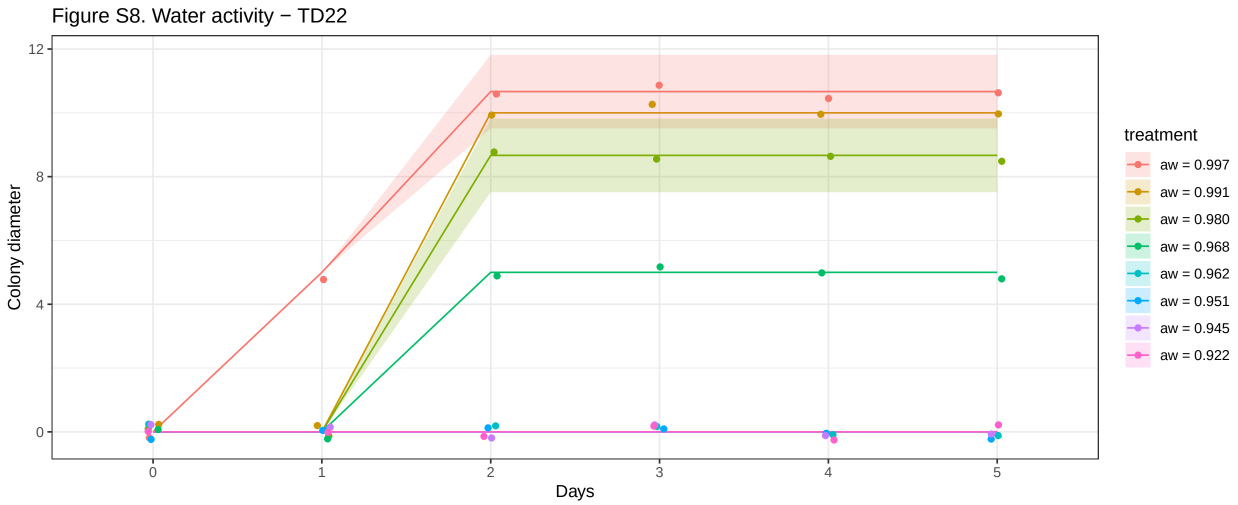


```
[1] "ANOVA results:"
                        Df Sum Sq Mean Sq F value  Pr(>F)    
data_stat_id$treatment  7   8825    1261     236 6.2e-15 ***
Residuals              16     85       5                    
---
Signif. codes:  0 '***' 0.001 '**' 0.01 '*' 0.05 '.' 0.1 ' ' 1
[1] ""
[1] "############"
[1] ""
[1] "Dunnett-test results:"

  Dunnett's test for comparing several treatments with a control :  
    95% family-wise confidence level

$`aw = 0.997`
                            diff   lwr.ci    upr.ci    pval    
aw = 0.991-aw = 0.997  -7.6667 -13.178  -2.1549  0.0050 ** 
aw = 0.980-aw = 0.997 -13.0000 -18.512  -7.4883 2.1e-05 ***
aw = 0.968-aw = 0.997 -27.6667 -33.178 -22.1549 8.8e-12 ***
aw = 0.962-aw = 0.997 -47.6667 -53.178 -42.1549 < 2e-16 ***
aw = 0.951-aw = 0.997 -47.6667 -53.178 -42.1549 < 2e-16 ***
aw = 0.945-aw = 0.997 -47.6667 -53.178 -42.1549 < 2e-16 ***
aw = 0.922-aw = 0.997 -47.6667 -53.178 -42.1549 < 2e-16 ***

---
Signif. codes:  0 '***' 0.001 '**' 0.01 '*' 0.05 '.' 0.1 ' ' 1

[1] ""
[1] "################################"
[1] "################################"
[1] ""
[1] "TD25"
```

Figure S9. Water activity − TD25

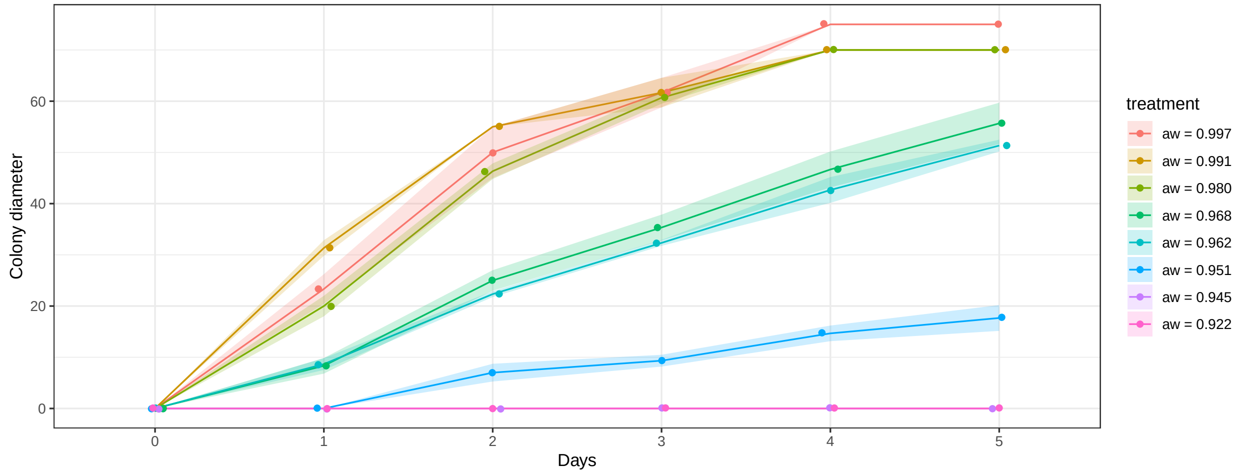


```
[1] "ANOVA results:"
                        Df Sum Sq Mean Sq F value Pr(>F)
data_stat_id$treatment   7 320055   45722    3670 <2e-16 ***
Residuals               16    199      12
---
Signif. codes:  0 ’***’ 0.001 ’**’ 0.01 ’*’ 0.05 ’.’ 0.1 ’ ’ 1
[1] ""
[1] "############"
[1] ""
[1] "Dunnett-test results:"

  Dunnett’s test for comparing several treatments with a control :
    95% family-wise confidence level

$‘aw = 0.997‘
                          diff   lwr.ci   upr.ci    pval
aw = 0.991-aw = 0.997     3.00   -5.424   11.424  0.8285
aw = 0.980-aw = 0.997   -18.00  -26.424   -9.576 8.2e-05 ***
aw = 0.968-aw = 0.997  -114.00 -122.424 -105.576 < 2e-16 ***
aw = 0.962-aw = 0.997  -127.67 -136.091 -119.243 < 2e-16 ***
aw = 0.951-aw = 0.997  -236.33 -244.757 -227.909 < 2e-16 ***
aw = 0.945-aw = 0.997  -285.00 -293.424 -276.576 < 2e-16 ***
aw = 0.922-aw = 0.997  -285.00 -293.424 -276.576 < 2e-16 ***

---
Signif. codes:  0 ’***’ 0.001 ’**’ 0.01 ’*’ 0.05 ’.’ 0.1 ’ ’ 1

[1] ""
[1] "################################"
[1] "################################"
[1] ""
[1] "TD4"
```

Figure S10. Water activity − TD4

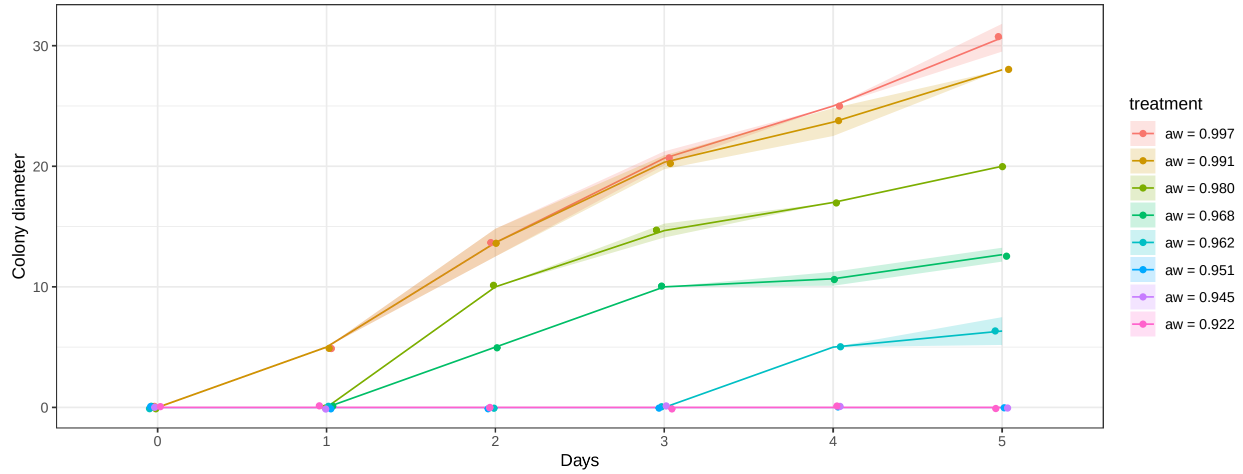


```
[1] "ANOVA results:"
                       Df Sum Sq Mean Sq F value Pr(>F)
data_stat_id$treatment  7  34860    4980    6291 <2e-16 ***
Residuals              16     13       1
---
Signif. codes:  0 '***' 0.001 '**' 0.01 '*' 0.05 '.' 0.1 ' ' 1
[1] ""
[1] "############"
[1] ""
[1] "Dunnett-test results:"

  Dunnett's test for comparing several treatments with a control :
    95% family-wise confidence level

$`aw = 0.997`
                             diff    lwr.ci    upr.ci    pval
aw = 0.991-aw = 0.997   -4.3333   -6.4569   -2.2098 9.4e-05 ***
aw = 0.980-aw = 0.997  -33.3333  -35.4569  -31.2098 < 2e-16 ***
aw = 0.968-aw = 0.997  -56.6667  -58.7902  -54.5431 < 2e-16 ***
aw = 0.962-aw = 0.997  -83.6667  -85.7902  -81.5431 < 2e-16 ***
aw = 0.951-aw = 0.997  -95.0000  -97.1235  -92.8765 < 2e-16 ***
aw = 0.945-aw = 0.997  -95.0000  -97.1235  -92.8765 < 2e-16 ***
aw = 0.922-aw = 0.997  -95.0000  -97.1235  -92.8765 < 2e-16 ***

---
Signif. codes:  0 '***' 0.001 '**' 0.01 '*' 0.05 '.' 0.1 ' ' 1

[1] ""
[1] "################################"
[1] "################################"
[1] ""
[1] "TD5"
```

Figure S11. Water activity − TD5

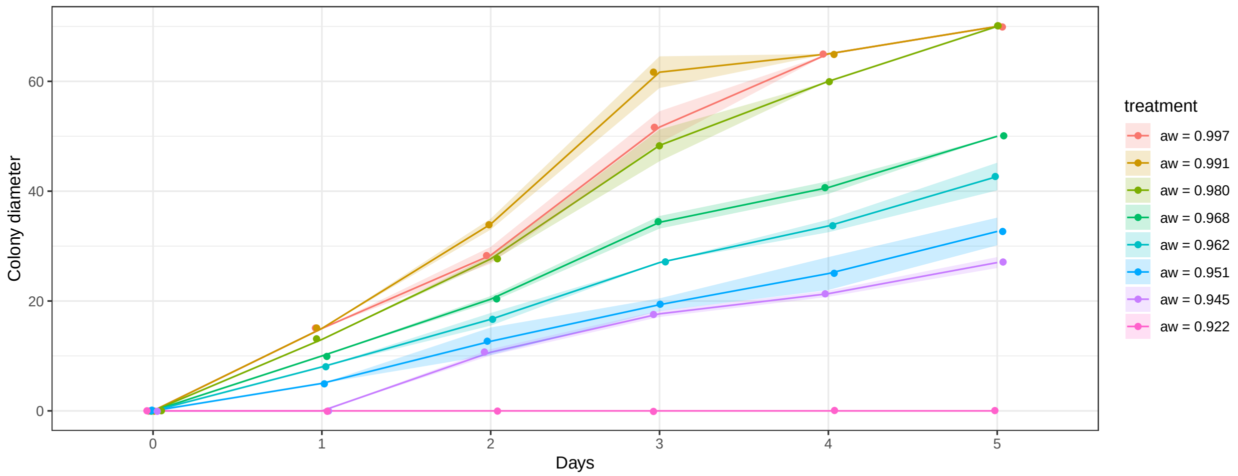


```
[1] "ANOVA results:"
                        Df Sum Sq Mean Sq F value Pr(>F)
data_stat_id$treatment  7 154333   22048    2043 <2e-16 ***
Residuals              16    173      11
---
Signif. codes:  0 '***' 0.001 '**' 0.01 '*' 0.05 '.' 0.1 ' ' 1
[1] ""
[1] "############"
[1] ""
[1] "Dunnett-test results:"

  Dunnett's test for comparing several treatments with a control :
    95% family-wise confidence level

$`aw = 0.997`
                              diff     lwr.ci     upr.ci     pval
aw = 0.991-aw = 0.997   15.667     7.8263    23.5070 0.00022 ***
aw = 0.980-aw = 0.997  -11.000   -18.8403    -3.1597 0.00477 **
aw = 0.968-aw = 0.997  -74.667   -82.5070   -66.8263 < 2e-16 ***
aw = 0.962-aw = 0.997 -102.000 -109.8403   -94.1597 < 2e-16 ***
aw = 0.951-aw = 0.997 -135.333 -143.1737 -127.4930 < 2e-16 ***
aw = 0.945-aw = 0.997 -153.333 -161.1737 -145.4930 < 2e-16 ***
aw = 0.922-aw = 0.997 -230.000 -237.8403 -222.1597 < 2e-16 ***

---
Signif. codes:  0 '***' 0.001 '**' 0.01 '*' 0.05 '.' 0.1 ' ' 1

[1] ""
[1] "################################"
[1] "################################"
[1] ""
[1] "TD6"
```

Figure S12. Water activity − TD6

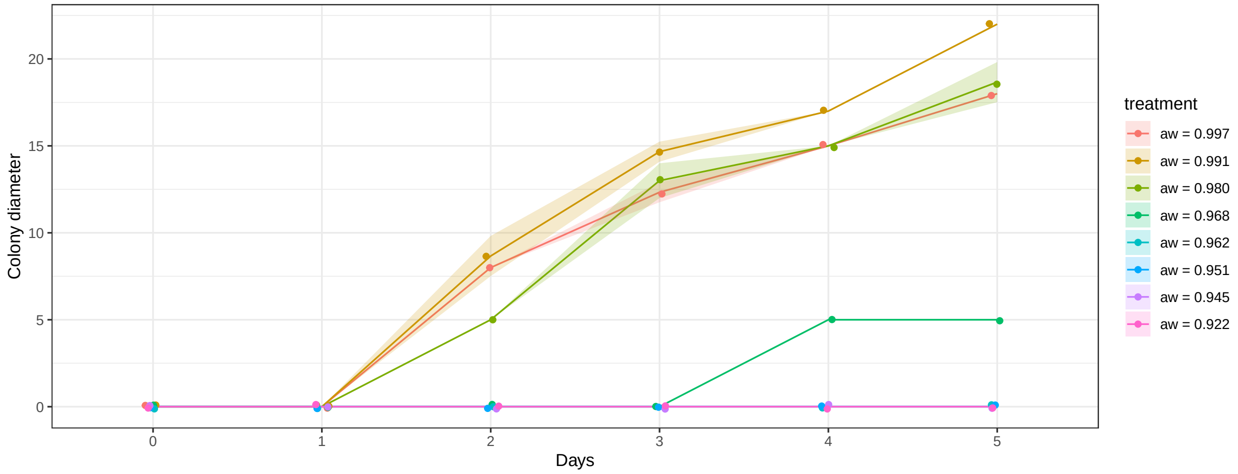


```
[1] "ANOVA results:"
                        Df Sum Sq Mean Sq F value Pr(>F)
data_stat_id$treatment  7  16705    2386   19092 <2e-16 ***
Residuals              16      2       0
---
Signif. codes:  0 '***' 0.001 '**' 0.01 '*' 0.05 '.' 0.1 ' ' 1
[1] ""
[1] "############"
[1] ""
[1] "Dunnett-test results:"

  Dunnett's test for comparing several treatments with a control :
    95% family-wise confidence level

$`aw = 0.997`
                           diff    lwr.ci     upr.ci     pval
aw = 0.991-aw = 0.997   9.0000    8.1562    9.84381 < 2e-16 ***
aw = 0.980-aw = 0.997  -1.6667   -2.5105   -0.82286 0.00015 ***
aw = 0.968-aw = 0.997 -43.3333 -44.1771 -42.48952 < 2e-16 ***
aw = 0.962-aw = 0.997 -53.3333 -54.1771 -52.48952 < 2e-16 ***
aw = 0.951-aw = 0.997 -53.3333 -54.1771 -52.48952 < 2e-16 ***
aw = 0.945-aw = 0.997 -53.3333 -54.1771 -52.48952 < 2e-16 ***
aw = 0.922-aw = 0.997 -53.3333 -54.1771 -52.48952 < 2e-16 ***

---
Signif. codes:  0 '***' 0.001 '**' 0.01 '*' 0.05 '.' 0.1 ' ' 1

[1] ""
[1] "################################"
[1] "################################"
[1] ""
[1] "TD8"
```

Figure S13. Water activity − TD8

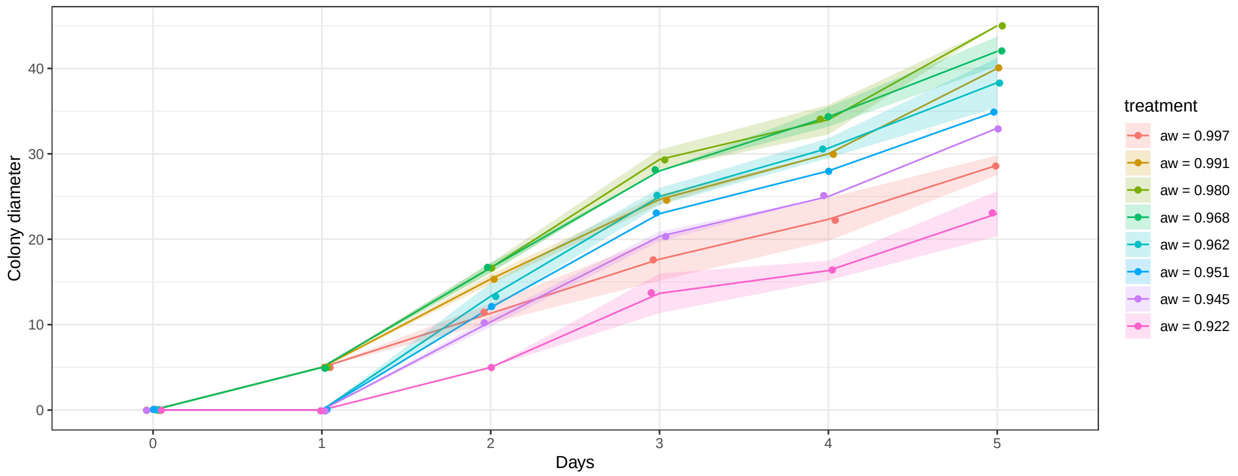


```
[1] "ANOVA results:"
                        Df Sum Sq Mean Sq F value  Pr(>F)
data_stat_id$treatment  7  11905    1701     292 1.2e-15 ***
Residuals              16     93       6
---
Signif. codes:  0 '***' 0.001 '**' 0.01 '*' 0.05 '.' 0.1 ' ' 1
[1] ""
[1] "############"
[1] ""
[1] "Dunnett-test results:"

  Dunnett's test for comparing several treatments with a control :
    95% family-wise confidence level

$`aw = 0.997`
                           diff    lwr.ci   upr.ci    pval
aw = 0.991-aw = 0.997  30.0000  24.2357  35.764 2.3e-14 ***
aw = 0.980-aw = 0.997  45.0000  39.2357  50.764 < 2e-16 ***
aw = 0.968-aw = 0.997  41.0000  35.2357  46.764 < 2e-16 ***
aw = 0.962-aw = 0.997  22.3333  16.5690  28.098 2.1e-09 ***
aw = 0.951-aw = 0.997  13.0000   7.2357  18.764 2.9e-05 ***
aw = 0.945-aw = 0.997   3.6667  -2.0977   9.431  0.3244
aw = 0.922-aw = 0.997 -27.0000 -32.7643 -21.236 2.1e-13 ***

---
Signif. codes:  0 '***' 0.001 '**' 0.01 '*' 0.05 '.' 0.1 ' ' 1

[1] ""
[1] "################################"
[1] "################################"
[1] ""
[1] "TD9"
```

Figure S14. Water activity − TD9

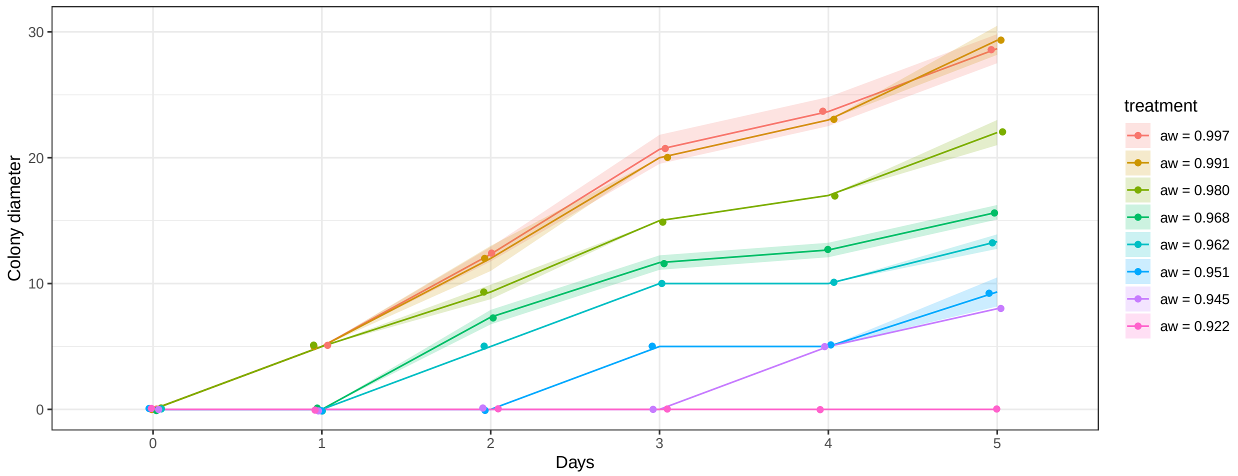


```
[1] "ANOVA results:"
                      Df Sum Sq Mean Sq F value Pr(>F)
data_stat_id$treatment  7  24954    3565    1901 <2e-16 ***
Residuals              16     30       2
---
Signif. codes:  0 ’***’ 0.001 ’**’ 0.01 ’*’ 0.05 ’.’ 0.1 ’ ’ 1
[1] ""
[1] "############"
[1] ""
[1] "Dunnett-test results:"

  Dunnett’s test for comparing several treatments with a control :
    95% family-wise confidence level

$‘aw = 0.997‘
                           diff    lwr.ci    upr.ci   pval
aw = 0.991-aw = 0.997   -1.000  -4.2681   2.2681 0.9030
aw = 0.980-aw = 0.997  -22.000 -25.2681 -18.7319 <2e-16 ***
aw = 0.968-aw = 0.997  -43.000 -46.2681 -39.7319 <2e-16 ***
aw = 0.962-aw = 0.997  -52.000 -55.2681 -48.7319 <2e-16 ***
aw = 0.951-aw = 0.997  -71.000 -74.2681 -67.7319 <2e-16 ***
aw = 0.945-aw = 0.997  -77.333 -80.6014 -74.0653 <2e-16 ***
aw = 0.922-aw = 0.997  -90.333 -93.6014 -87.0653 <2e-16 ***

---
Signif. codes:  0 ’***’ 0.001 ’**’ 0.01 ’*’ 0.05 ’.’ 0.1 ’ ’ 1

[1] ""
[1] "################################"
[1] "################################"
[1] ""
```

Substrate

```
[1] "SZMC26543"
```

Figure S15. Substrate − SZMC26543

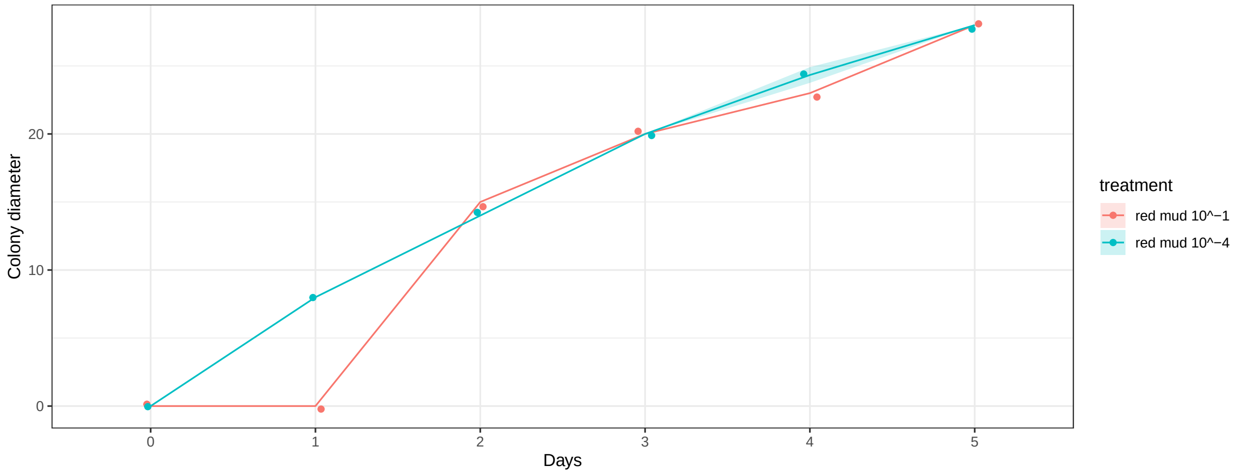


```
[1] "ANOVA results:"
                        Df Sum Sq Mean Sq F value  Pr(>F)
data_stat_id$treatment  1  104.2   104.2     625 1.5e-05 ***
Residuals               4    0.7     0.2
---
Signif. codes:  0 '***' 0.001 '**' 0.01 '*' 0.05 '.' 0.1 ' ' 1
[1] ""
[1] "############"
[1] ""
[1] "Dunnett-teszt results:"

  Dunnett's test for comparing several treatments with a control :
    95% family-wise confidence level

$`red mud 10^-1`
                              diff lwr.ci upr.ci    pval
red mud 10^-4-red mud 10^-1 8.3333 7.4079 9.2588 1.5e-05 ***

---
Signif. codes:  0 '***' 0.001 '**' 0.01 '*' 0.05 '.' 0.1 ' ' 1

[1] ""
[1] "################################"
[1] "################################"
[1] ""
[1] "T398A"
```

Figure S16. Substrate − T398A

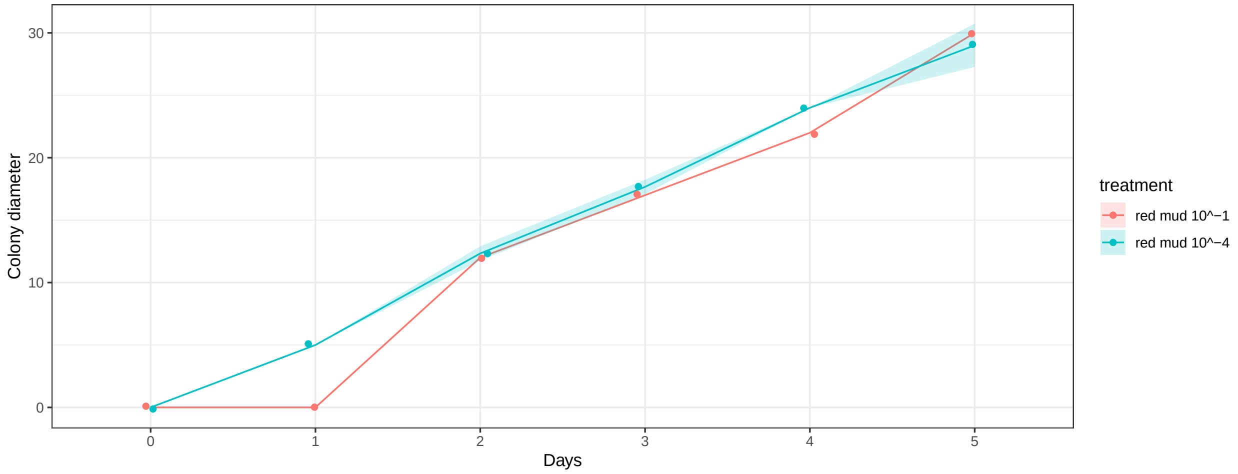


```
[1] "ANOVA results:"
                      Df Sum Sq Mean Sq F value Pr(>F)
data_stat_id$treatment  1   73.5    73.5      21   0.01 *
Residuals               4   14.0     3.5
---
Signif. codes:  0 '***' 0.001 '**' 0.01 '*' 0.05 '.' 0.1 ' ' 1
[1] ""
[1] "############"
[1] ""
[1] "Dunnett-teszt results:"

  Dunnett's test for comparing several treatments with a control :
    95% family-wise confidence level

$`red mud 10^-1`
                            diff lwr.ci upr.ci   pval
red mud 10^-4-red mud 10^-1    7 2.7589 11.241 0.0102 *

---
Signif. codes:  0 '***' 0.001 '**' 0.01 '*' 0.05 '.' 0.1 ' ' 1

[1] ""
[1] "################################"
[1] "################################"
[1] ""
[1] "T398B"
```

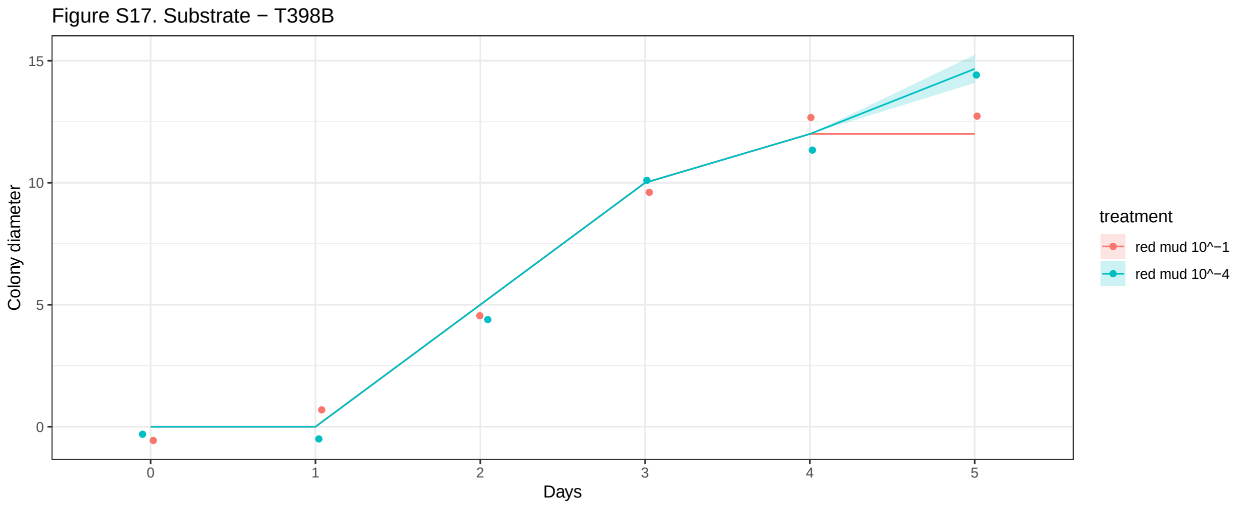


```
[1] "ANOVA results:"
                       Df Sum Sq Mean Sq F value Pr(>F)
data_stat_id$treatment  1  10.67   10.67      64 0.0013 **
Residuals               4   0.67    0.17
---
Signif. codes:  0 ’***’ 0.001 ’**’ 0.01 ’*’ 0.05 ’.’ 0.1 ’ ’ 1
[1] ""
[1] "############"
[1] ""
[1] "Dunnett-teszt results:"

  Dunnett’s test for comparing several treatments with a control :
    95% family-wise confidence level

$‘red mud 10^-1‘
                              diff lwr.ci upr.ci   pval
red mud 10^-4-red mud 10^-1 2.6667 1.7412 3.5921 0.0013 **

---
Signif. codes:  0 ’***’ 0.001 ’**’ 0.01 ’*’ 0.05 ’.’ 0.1 ’ ’ 1

[1] ""
[1] "################################"
[1] "################################"
[1] ""
[1] "T399"
```

Figure S18. Substrate − T399

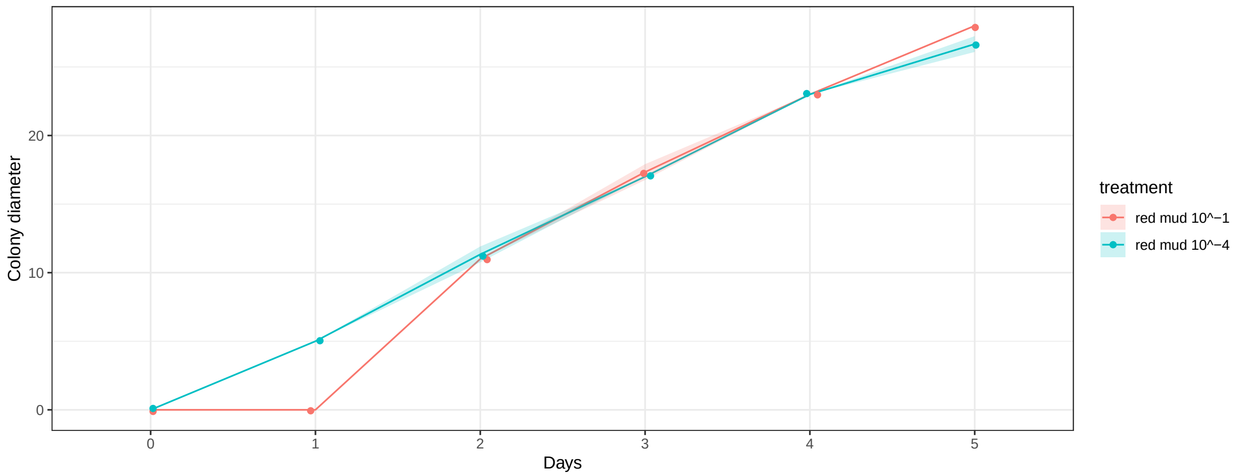


```
[1] "ANOVA results:"
                       Df Sum Sq Mean Sq F value  Pr(>F)
data_stat_id$treatment  1  20.17   20.17     121 0.00039 ***
Residuals               4   0.67    0.17
---
Signif. codes:  0 '***' 0.001 '**' 0.01 '*' 0.05 '.' 0.1 ' ' 1
[1] ""
[1] "############"
[1] ""
[1] "Dunnett-teszt results:"

  Dunnett's test for comparing several treatments with a control :
    95% family-wise confidence level

$`red mud 10^-1`
                              diff lwr.ci upr.ci    pval
red mud 10^-4-red mud 10^-1 3.6667 2.7412 4.5921 0.00039 ***

---
Signif. codes:  0 '***' 0.001 '**' 0.01 '*' 0.05 '.' 0.1 ' ' 1

[1] ""
[1] "################################"
[1] "################################"
[1] ""
[1] "T401A"
```

Figure S19. Substrate − T401A

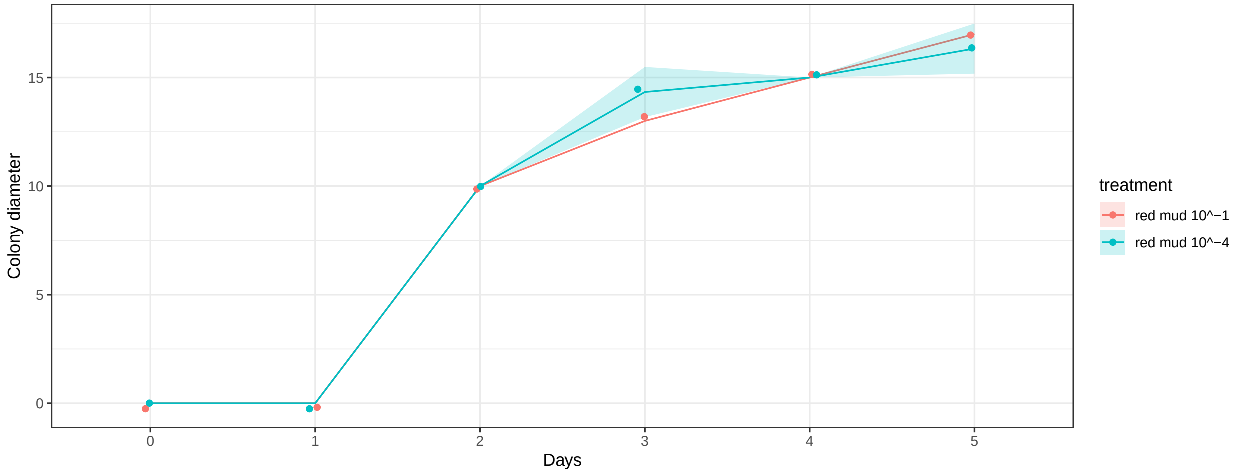


```
[1] "ANOVA results:"
                        Df Sum Sq Mean Sq F value Pr(>F)
data_stat_id$treatment  1  0.667   0.667       1   0.37
Residuals               4  2.667   0.667
[1] ""
[1] "############"
[1] ""
[1] "Dunnett-teszt results:"

  Dunnett's test for comparing several treatments with a control :
    95% family-wise confidence level

$`red mud 10^-1`
                               diff   lwr.ci upr.ci   pval
red mud 10^-4-red mud 10^-1 0.66667 -1.1843 2.5176 0.3739

---
Signif. codes:  0 '***' 0.001 '**' 0.01 '*' 0.05 '.' 0.1 ' ' 1

[1] ""
[1] "################################"
[1] "################################"
[1] ""
[1] "T401B"
```

Figure S20. Substrate − T401B

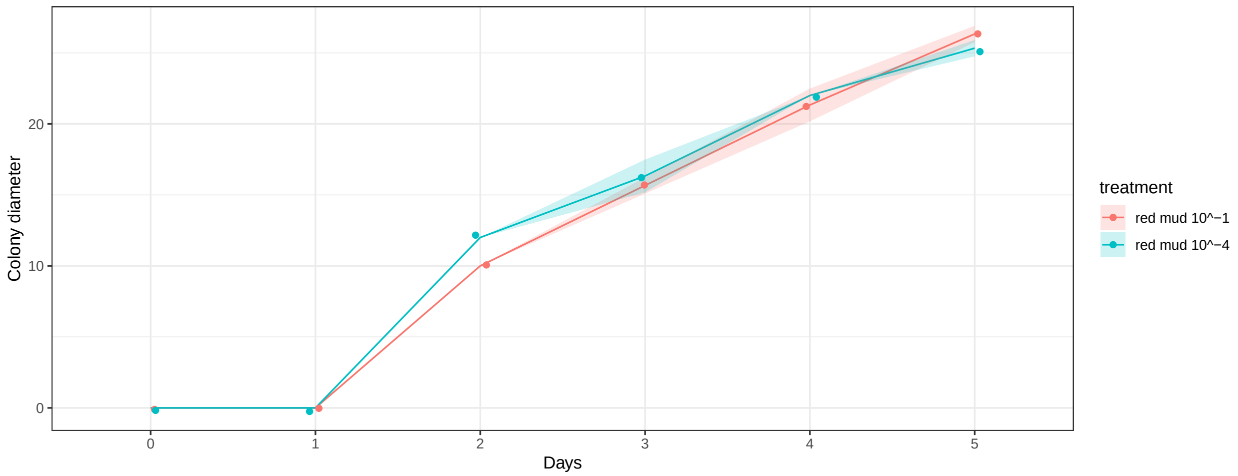


```
[1] "ANOVA results:"
                       Df Sum Sq Mean Sq F value Pr(>F)
data_stat_id$treatment  1   8.17    8.17     3.5   0.13
Residuals               4   9.33    2.33
[1] ""
[1] "############"
[1] ""
[1] "Dunnett-teszt results:"

  Dunnett's test for comparing several treatments with a control :
    95% family-wise confidence level

$`red mud 10^-1`
                              diff  lwr.ci upr.ci   pval
red mud 10^-4-red mud 10^-1 2.3333 -1.1295 5.7962 0.1347

---
Signif. codes:  0 '***' 0.001 '**' 0.01 '*' 0.05 '.' 0.1 ' ' 1

[1] ""
[1] "################################"
[1] "################################"
[1] ""
[1] "TD12"
```

Figure S21. Substrate − TD12

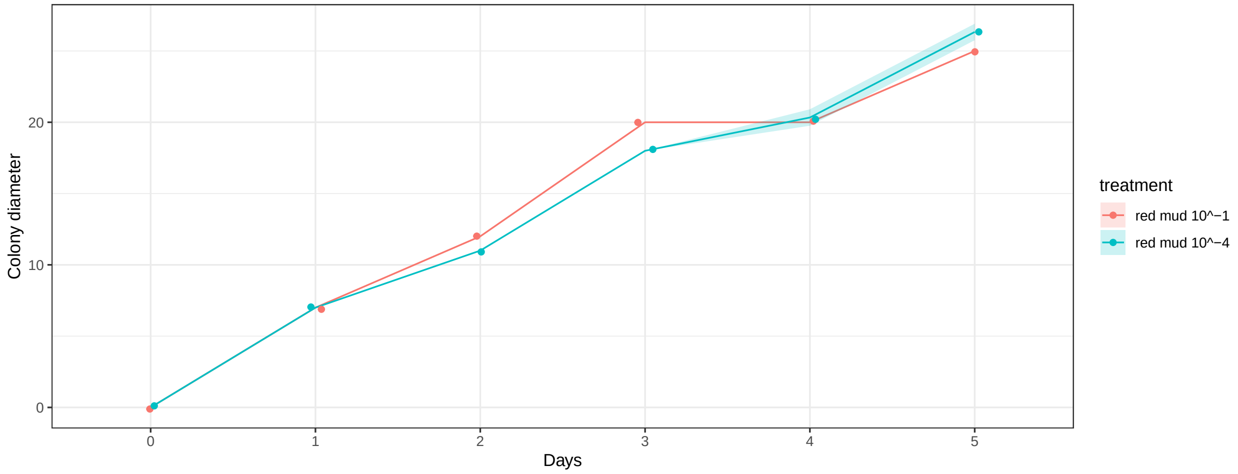


```
[1] "ANOVA results:"
                        Df Sum Sq Mean Sq F value Pr(>F)
data_stat_id$treatment  1   2.67   2.667       4   0.12
Residuals               4   2.67   0.667
[1] ""
[1] "############"
[1] ""
[1] "Dunnett-teszt results:"

  Dunnett's test for comparing several treatments with a control :
    95% family-wise confidence level

$`red mud 10^-1`
                               diff   lwr.ci   upr.ci    pval
red mud 10^-4-red mud 10^-1 -1.3333 -3.1843 0.51763 0.1161

---
Signif. codes:  0 '***' 0.001 '**' 0.01 '*' 0.05 '.' 0.1 ' ' 1

[1] ""
[1] "################################"
[1] "################################"
[1] ""
[1] "TD16"
```

Figure S22. Substrate − TD16

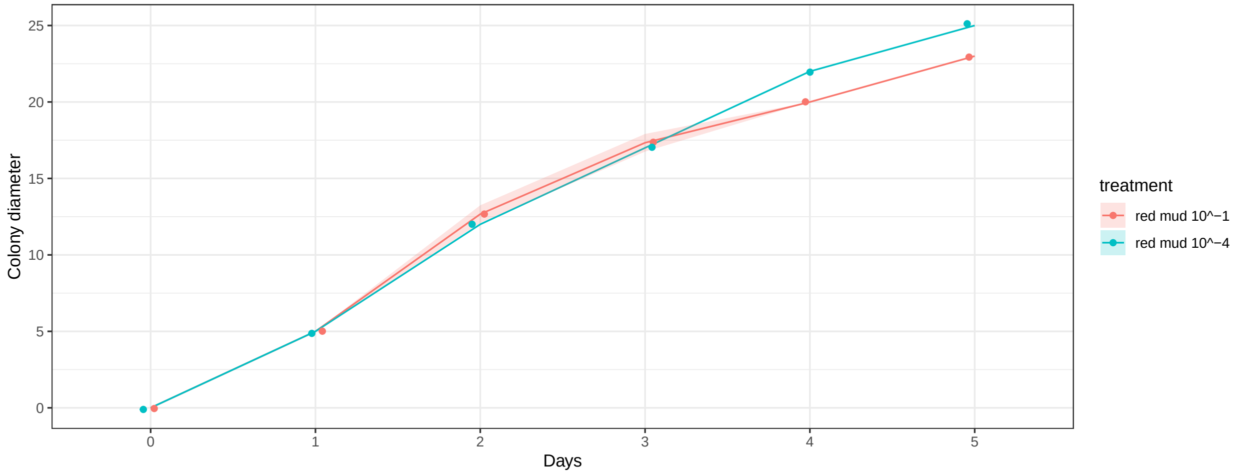


```
[1] "ANOVA results:"
                       Df Sum Sq Mean Sq F value Pr(>F)
data_stat_id$treatment  1   13.5    13.5 4.5e+28 <2e-16 ***
Residuals               4    0.0     0.0
---
Signif. codes:  0 '***' 0.001 '**' 0.01 '*' 0.05 '.' 0.1 ' ' 1
[1] ""
[1] "############"
[1] ""
[1] "Dunnett-teszt results:"

  Dunnett's test for comparing several treatments with a control :
    95% family-wise confidence level

$`red mud 10^-1`
                            diff lwr.ci upr.ci    pval
red mud 10^-4-red mud 10^-1    3      3      3 <2e-16 ***

---
Signif. codes:  0 '***' 0.001 '**' 0.01 '*' 0.05 '.' 0.1 ' ' 1

[1] ""
[1] "################################"
[1] "################################"
[1] ""
[1] "TD22"
```

Figure S23. Substrate − TD22

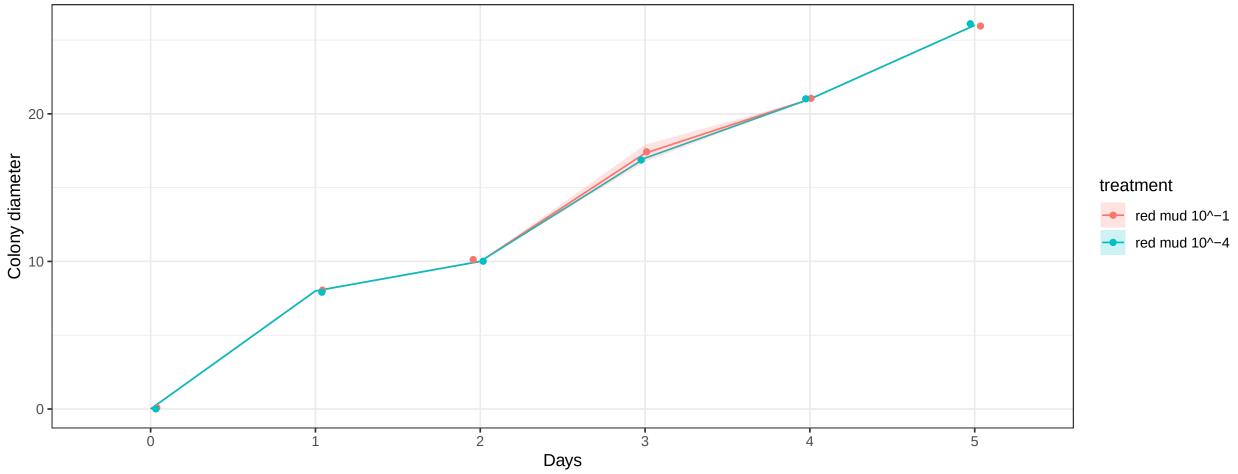


```
[1] "ANOVA results:"
                        Df Sum Sq Mean Sq F value Pr(>F)
data_stat_id$treatment  1  0.167   0.167       1   0.37
Residuals               4  0.667   0.167
[1] ""
[1] "############"
[1] ""
[1] "Dunnett-teszt results:"

  Dunnett's test for comparing several treatments with a control :
    95% family-wise confidence level

$`red mud 10^-1`
                                  diff   lwr.ci  upr.ci    pval
red mud 10^-4-red mud 10^-1 -0.33333 -1.2588 0.59215 0.3739

---
Signif. codes:  0 '***' 0.001 '**' 0.01 '*' 0.05 '.' 0.1 ' ' 1

[1] ""
[1] "################################"
[1] "################################"
[1] ""
[1] "TD25"
```

Figure S24. Substrate − TD25

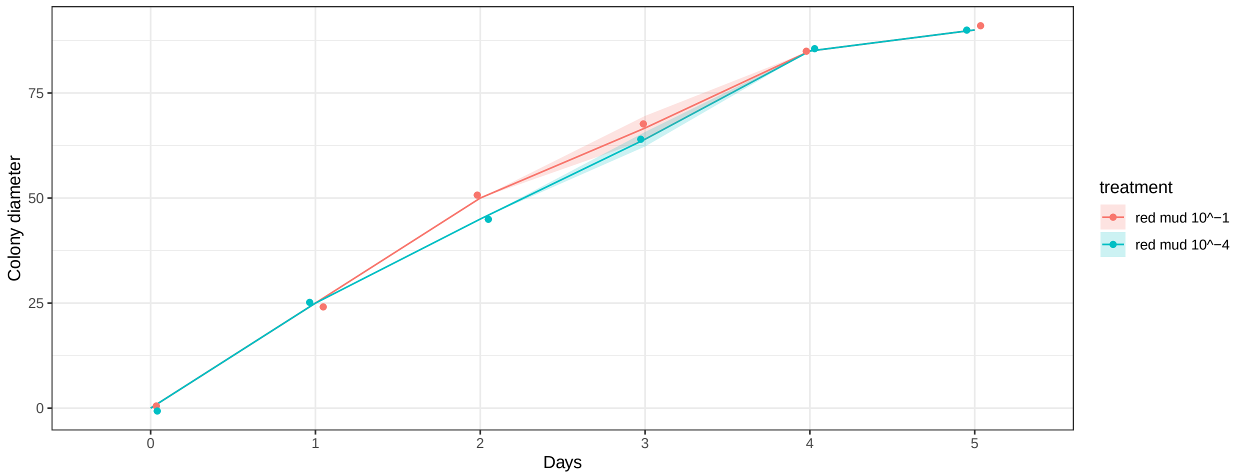


```
[1] "ANOVA results:"
                       Df Sum Sq Mean Sq F value Pr(>F)
data_stat_id$treatment  1   88.2    88.2    15.6  0.017 *
Residuals               4   22.7     5.7
---
Signif. codes:  0 '***' 0.001 '**' 0.01 '*' 0.05 '.' 0.1 ' ' 1
[1] ""
[1] "############"
[1] ""
[1] "Dunnett-teszt results:"

  Dunnett's test for comparing several treatments with a control :
    95% family-wise confidence level

$`red mud 10^-1`
                                 diff  lwr.ci  upr.ci   pval
red mud 10^-4-red mud 10^-1 -7.6667 -13.063 -2.2702 0.0169 *

---
Signif. codes:  0 '***' 0.001 '**' 0.01 '*' 0.05 '.' 0.1 ' ' 1

[1] ""
[1] "################################"
[1] "################################"
[1] ""
[1] "TD4"
```

Figure S25. Substrate − TD4

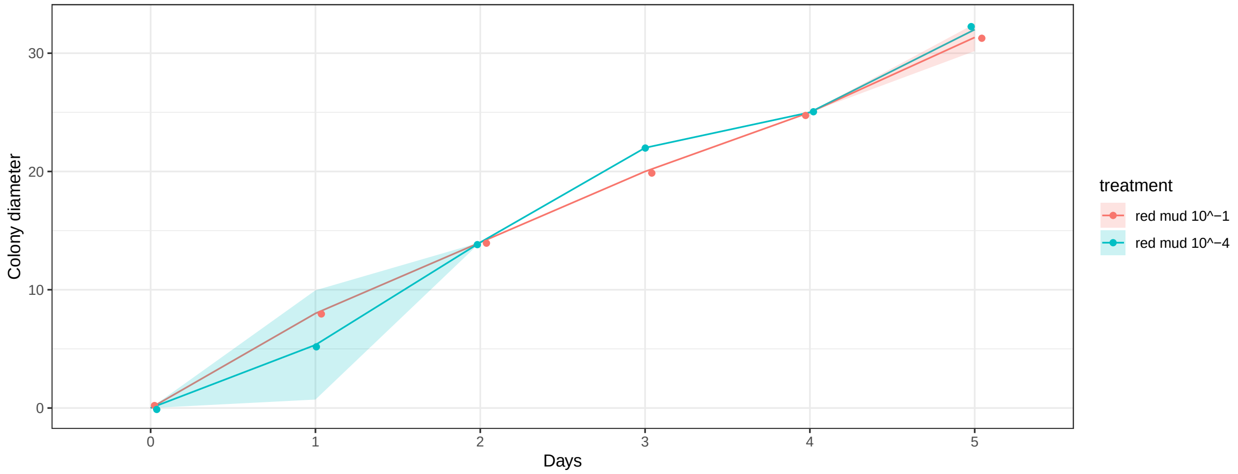


```
[1] "ANOVA results:"
                        Df Sum Sq Mean Sq F value Pr(>F)
data_stat_id$treatment  1    0.0     0.0       0      1
Residuals               4   45.3    11.3
[1] ""
[1] "############"
[1] ""
[1] "Dunnett-teszt results:"

  Dunnett's test for comparing several treatments with a control :
    95% family-wise confidence level

$`red mud 10^-1`
                            diff  lwr.ci upr.ci   pval
red mud 10^-4-red mud 10^-1    0 -7.6317 7.6317 1.0000

---
Signif. codes:  0 '***' 0.001 '**' 0.01 '*' 0.05 '.' 0.1 ' ' 1

[1] ""
[1] "################################"
[1] "################################"
[1] ""
[1] "TD5"
```

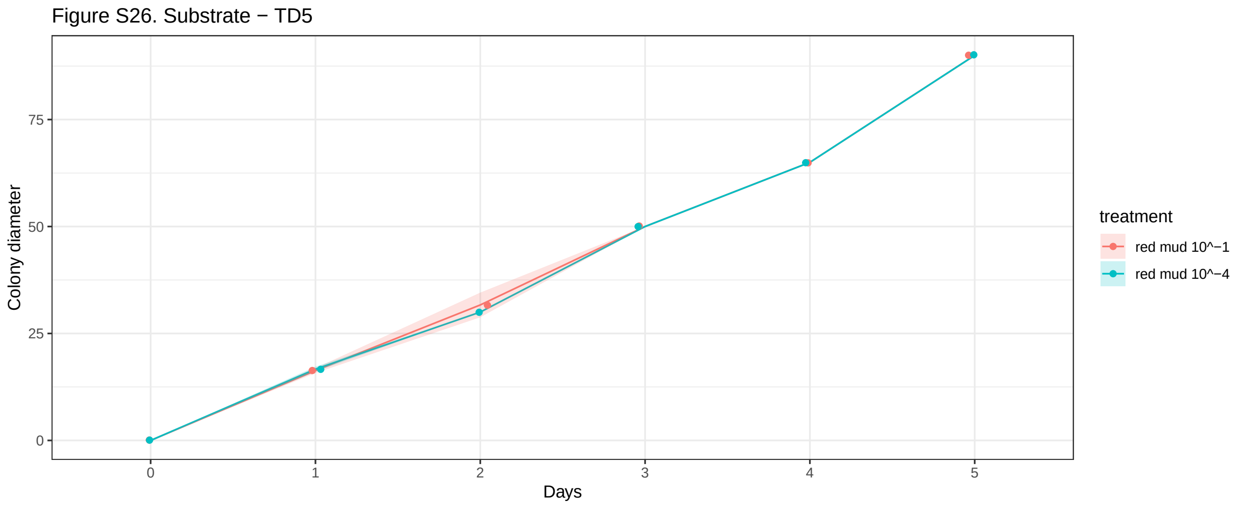


```
[1] "ANOVA results:"
                      Df Sum Sq Mean Sq F value Pr(>F)
data_stat_id$treatment  1   2.67    2.67    0.73   0.44
Residuals               4  14.67    3.67
[1] ""
[1] "############"
[1] ""
[1] "Dunnett-teszt results:"

  Dunnett's test for comparing several treatments with a control :
    95% family-wise confidence level

$`red mud 10^-1`
                               diff   lwr.ci upr.ci   pval
red mud 10^-4-red mud 10^-1 -1.3333 -5.6742 3.0076 0.4418

---
Signif. codes:  0 '***' 0.001 '**' 0.01 '*' 0.05 '.' 0.1 ' ' 1

[1] ""
[1] "################################"
[1] "################################"
[1] ""
[1] "TD6"
```

Figure S27. Substrate − TD6

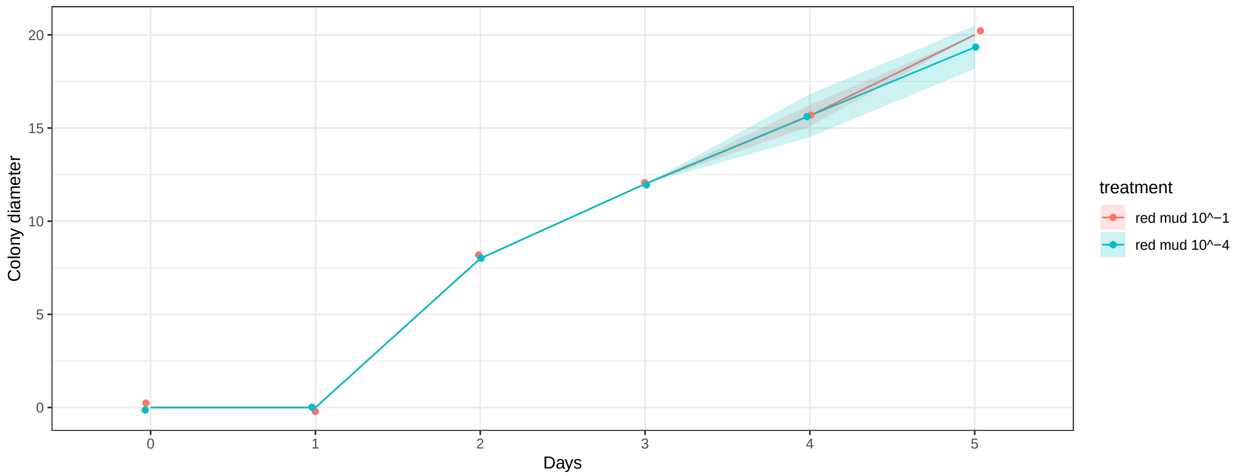


```
[1] "ANOVA results:"
                       Df Sum Sq Mean Sq F value Pr(>F)
data_stat_id$treatment  1  0.667   0.667       4   0.12
Residuals               4  0.667   0.167
[1] ""
[1] "############"
[1] ""
[1] "Dunnett-teszt results:"

  Dunnett's test for comparing several treatments with a control :
    95% family-wise confidence level

$`red mud 10^-1`
                                diff  lwr.ci  upr.ci   pval
red mud 10^-4-red mud 10^-1 -0.66667 -1.5921 0.25882 0.1161

---
Signif. codes:  0 '***' 0.001 '**' 0.01 '*' 0.05 '.' 0.1 ' ' 1

[1] ""
[1] "################################"
[1] "################################"
[1] ""
[1] "TD8"
```

Figure S28. Substrate − TD8

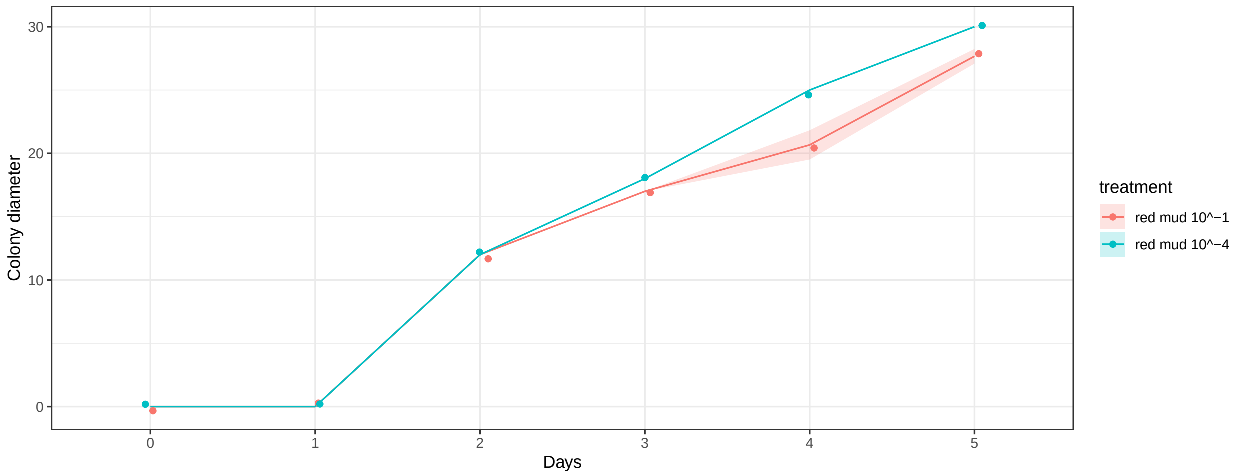


```
[1] "ANOVA results:"
                        Df Sum Sq Mean Sq F value  Pr(>F)    
data_stat_id$treatment  1   88.2    88.2    75.6 0.00096 ***
Residuals               4    4.7     1.2                    
---
Signif. codes:  0 ’***’ 0.001 ’**’ 0.01 ’*’ 0.05 ’.’ 0.1 ’ ’ 1
[1] ""
[1] "############"
[1] ""
[1] "Dunnett-teszt results:"

  Dunnett’s test for comparing several treatments with a control :  
    95% family-wise confidence level

$‘red mud 10^-1‘
                              diff lwr.ci upr.ci    pval    
red mud 10^-4-red mud 10^-1 7.6667 5.2181 10.115 0.00096 ***

---
Signif. codes:  0 ’***’ 0.001 ’**’ 0.01 ’*’ 0.05 ’.’ 0.1 ’ ’ 1

[1] ""
[1] "################################"
[1] "################################"
[1] ""
[1] "TD9"
```

Figure S29. Substrate − TD9

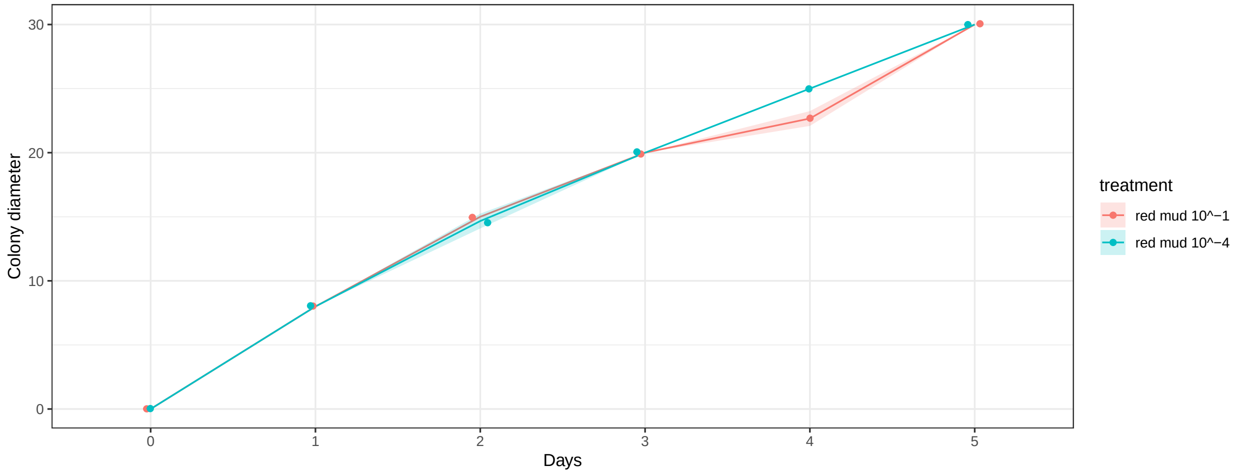


```
[1] "ANOVA results:"
                       Df Sum Sq Mean Sq F value Pr(>F)
data_stat_id$treatment  1   6.00    6.00      18  0.013 *
Residuals               4   1.33    0.33
---
Signif. codes:  0 '***' 0.001 '**' 0.01 '*' 0.05 '.' 0.1 ' ' 1
[1] ""
[1] "############"
[1] ""
[1] "Dunnett-teszt results:"

  Dunnett's test for comparing several treatments with a control :
    95% family-wise confidence level

$`red mud 10^-1`
                            diff  lwr.ci upr.ci   pval
red mud 10^-4-red mud 10^-1    2 0.69117 3.3088 0.0132 *

---
Signif. codes:  0 '***' 0.001 '**' 0.01 '*' 0.05 '.' 0.1 ' ' 1

[1] ""
[1] "################################"
[1] "################################"
[1] ""
```

pH

```
[1] "T398A"
```

Figure S30. pH – T398A

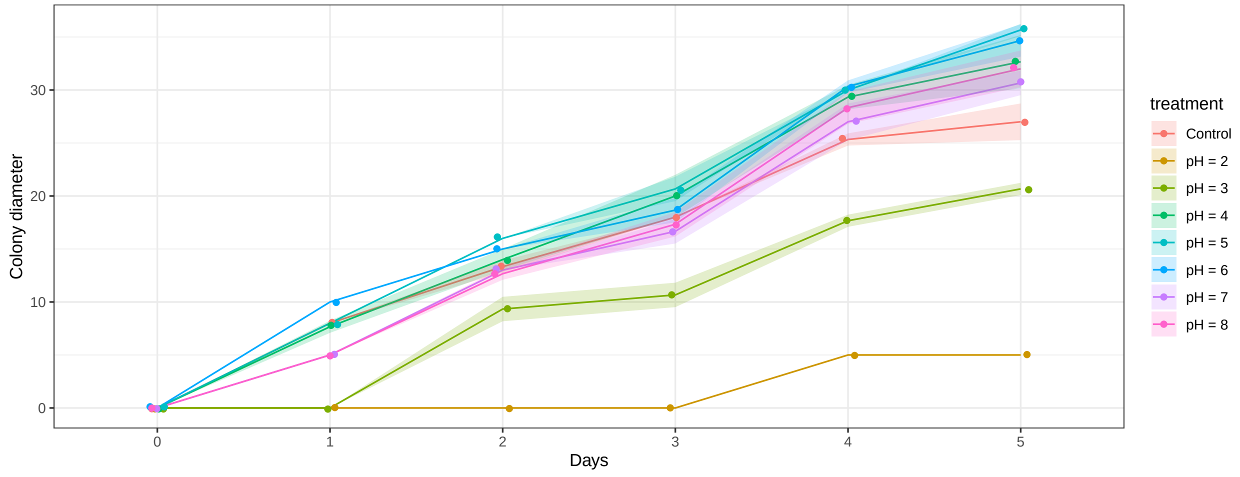


```
[1] "ANOVA results:"
                        Df  Sum Sq  Mean Sq  F value   Pr(>F)
data_stat_id$treatment   7    9521     1360      172 1.7e-12 ***
Residuals               14     111        8
---
Signif. codes:  0 '***' 0.001 '**' 0.01 '*' 0.05 '.' 0.1 ' ' 1
[1] ""
[1] "############"
[1] ""
[1] "Dunnett-test results:"

  Dunnett's test for comparing several treatments with a control :
    95% family-wise confidence level

$Control
                    diff   lwr.ci   upr.ci     pval
pH = 2-Control -61.66667 -71.3745 -51.9588 6.2e-15 ***
pH = 3-Control -33.33333 -40.1978 -26.4688 1.1e-11 ***
pH = 4-Control  12.00000   5.1355  18.8645 0.00075 ***
pH = 5-Control  18.66667  11.8022  25.5312 5.3e-06 ***
pH = 6-Control  17.00000  10.1355  23.8645 1.4e-05 ***
pH = 7-Control   0.66667  -6.1978   7.5312 0.99983
pH = 8-Control   3.66667  -3.1978  10.5312 0.48647

---
Signif. codes:  0 '***' 0.001 '**' 0.01 '*' 0.05 '.' 0.1 ' ' 1

[1] ""
[1] "################################"
[1] "################################"
[1] ""
[1] "T398B"
```

Figure S31. pH − T398B

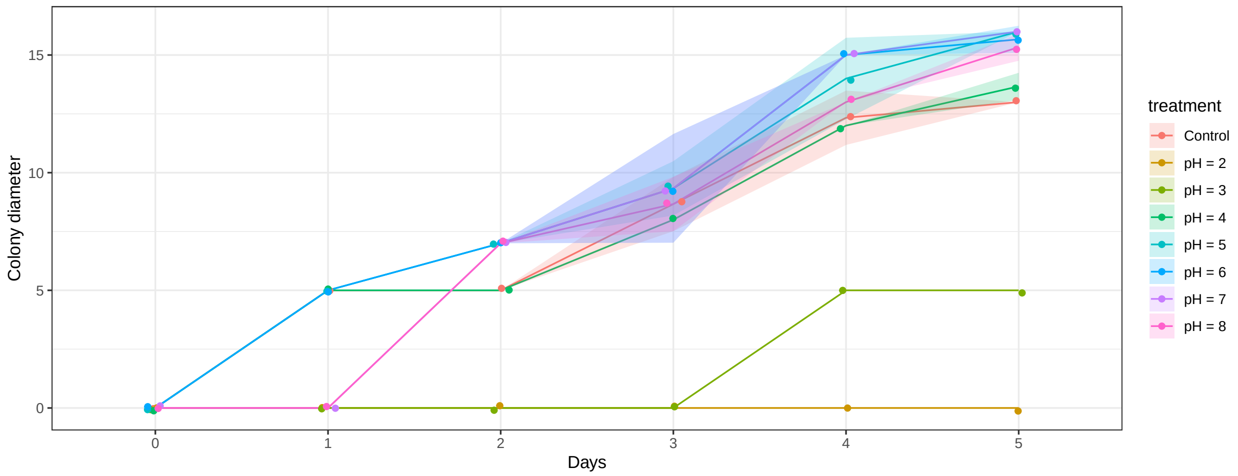


```
[1] "ANOVA results:"
                       Df Sum Sq Mean Sq F value  Pr(>F)
data_stat_id$treatment  7   5415     774     246 1.5e-13 ***
Residuals              14     44       3
---
Signif. codes:  0 '***' 0.001 '**' 0.01 '*' 0.05 '.' 0.1 ' ' 1
[1] ""
[1] "############"
[1] ""
[1] "Dunnett-test results:"

  Dunnett's test for comparing several treatments with a control :
    95% family-wise confidence level

$Control
                   diff    lwr.ci   upr.ci    pval
pH = 2-Control -44.00000 -50.12127 -37.8787 2.3e-15 ***
pH = 3-Control -34.00000 -38.32839 -29.6716 < 2e-16 ***
pH = 4-Control  -0.33333  -4.66173   3.9951 0.99996
pH = 5-Control   7.33333   3.00494  11.6617 0.00099 ***
pH = 6-Control   8.00000   3.67161  12.3284 0.00037 ***
pH = 7-Control   3.33333  -0.99506   7.6617 0.16953
pH = 8-Control   0.00000  -4.32839   4.3284 1.00000

---
Signif. codes:  0 '***' 0.001 '**' 0.01 '*' 0.05 '.' 0.1 ' ' 1

[1] ""
[1] "################################"
[1] "################################"
[1] ""
[1] "T399"
```

Figure S32. pH – T399

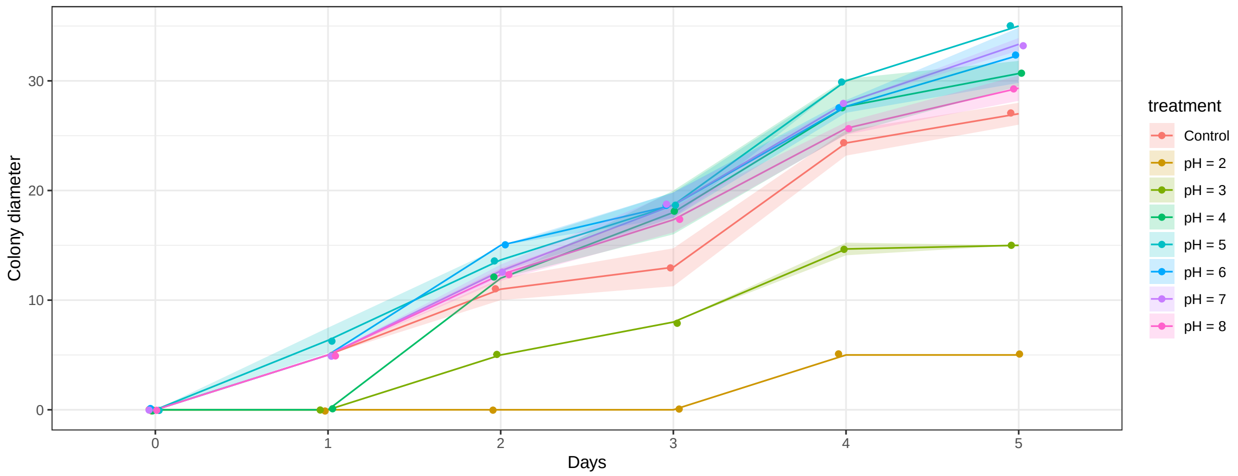


```
[1] "ANOVA results:"
                       Df Sum Sq Mean Sq F value  Pr(>F)    
data_stat_id$treatment  7  10590    1513     284 5.5e-14 ***
Residuals              14     75       5                    
---
Signif. codes:  0 ’***’ 0.001 ’**’ 0.01 ’*’ 0.05 ’.’ 0.1 ’ ’ 1
[1] ""
[1] "############"
[1] ""
[1] "Dunnett-test results:"

  Dunnett’s test for comparing several treatments with a control :  
    95% family-wise confidence level

$Control
                   diff    lwr.ci   upr.ci    pval    
pH = 2-Control -50.3333 -58.3074 -42.359 < 2e-16 ***
pH = 3-Control -37.6667 -43.3052 -32.028 2.2e-16 ***
pH = 4-Control   8.0000   2.3615  13.639  0.0046 ** 
pH = 5-Control  23.3333  17.6948  28.972 1.3e-09 ***
pH = 6-Control  18.3333  12.6948  23.972 3.3e-07 ***
pH = 7-Control  17.3333  11.6948  22.972 1.0e-06 ***
pH = 8-Control   9.3333   3.6948  14.972  0.0013 ** 

---
Signif. codes:  0 ’***’ 0.001 ’**’ 0.01 ’*’ 0.05 ’.’ 0.1 ’ ’ 1

[1] ""
[1] "################################"
[1] "################################"
[1] ""
[1] "T401A"
```

Figure S33. pH – T401A

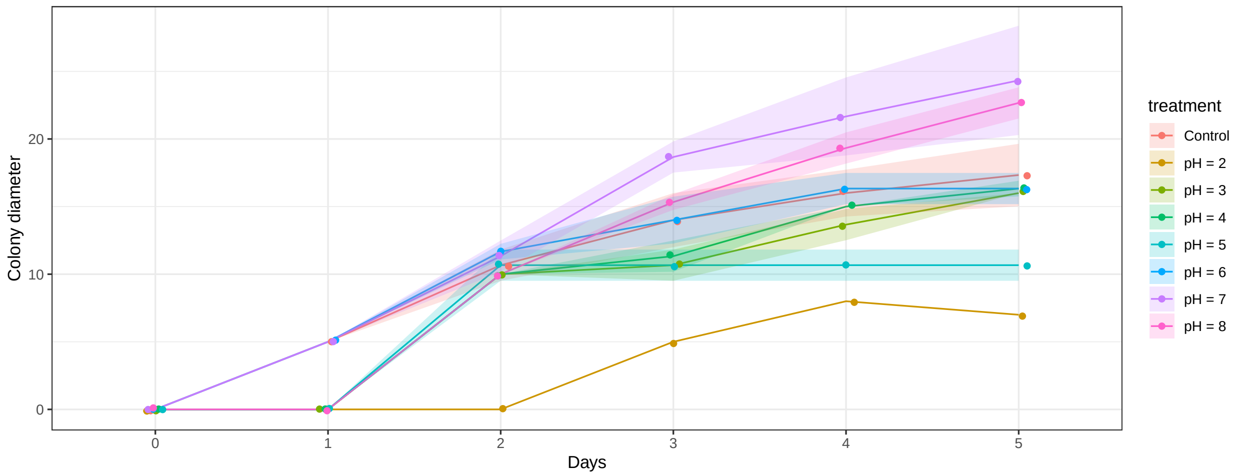


```
[1] "ANOVA results:"
                       Df Sum Sq Mean Sq F value  Pr(>F)    
data_stat_id$treatment  7   2888     413    53.8 4.7e-09 ***
Residuals              14    107       8                    
---
Signif. codes:  0 ’***’ 0.001 ’**’ 0.01 ’*’ 0.05 ’.’ 0.1 ’ ’ 1
[1] ""
[1] "############"
[1] ""
[1] "Dunnett-test results:"

  Dunnett’s test for comparing several treatments with a control :  
    95% family-wise confidence level

$Control
                    diff  lwr.ci   upr.ci    pval    
pH = 2-Control  -3.00000 -12.561   6.5605  0.8905    
pH = 3-Control -12.66667 -19.427  -5.9063  0.0004 ***
pH = 4-Control -10.33333 -17.094  -3.5730  0.0024 ** 
pH = 5-Control -20.33333 -27.094 -13.5730 4.6e-07 ***
pH = 6-Control   0.33333  -6.427   7.0937  1.0000    
pH = 7-Control  18.00000  11.240  24.7603 1.0e-05 ***
pH = 8-Control   4.33333  -2.427  11.0937  0.3128    

---
Signif. codes:  0 ’***’ 0.001 ’**’ 0.01 ’*’ 0.05 ’.’ 0.1 ’ ’ 1

[1] ""
[1] "################################"
[1] "################################"
[1] ""
[1] "T401B"
```

Figure S34. pH − T401B

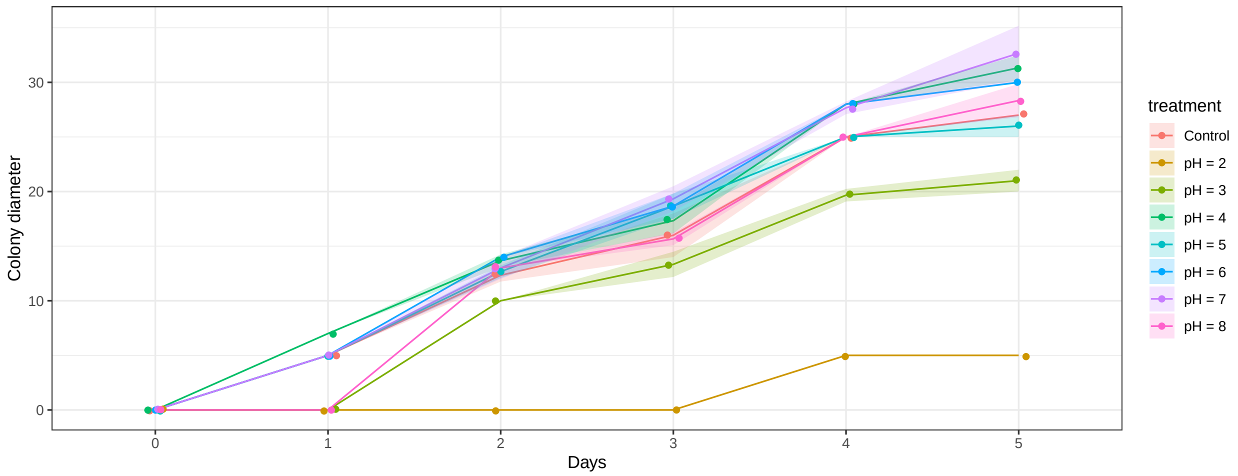


```
[1] "ANOVA results:"
                        Df Sum Sq Mean Sq F value  Pr(>F)    
data_stat_id$treatment  7   5664     809     221 3.1e-13 ***
Residuals              14     51       4                    
---
Signif. codes:  0 ’***’ 0.001 ’**’ 0.01 ’*’ 0.05 ’.’ 0.1 ’ ’ 1
[1] ""
[1] "############"
[1] ""
[1] "Dunnett-test results:"

  Dunnett’s test for comparing several treatments with a control :  
    95% family-wise confidence level

$Control
                    diff    lwr.ci    upr.ci    pval    
pH = 2-Control -55.3333 -61.9451 -48.7216 < 2e-16 ***
pH = 3-Control -21.3333 -26.0085 -16.6581 1.4e-10 ***
pH = 4-Control  12.0000   7.3248  16.6752 2.1e-05 ***
pH = 5-Control   2.0000  -2.6752   6.6752  0.6928    
pH = 6-Control  10.3333   5.6581  15.0085 6.1e-05 ***
pH = 7-Control  12.3333   7.6581  17.0085 7.6e-06 ***
pH = 8-Control  -3.3333  -8.0085   1.3419  0.2243    

---
Signif. codes:  0 ’***’ 0.001 ’**’ 0.01 ’*’ 0.05 ’.’ 0.1 ’ ’ 1

[1] ""
[1] "################################"
[1] "################################"
[1] ""
[1] "TD12"
```

Figure S35. pH – TD12

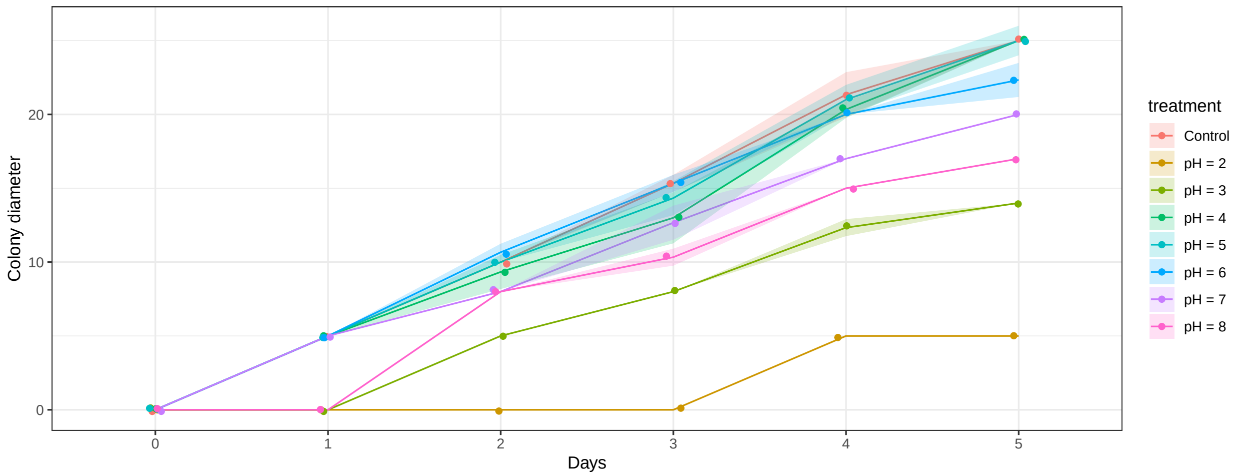


```
[1] "ANOVA results:"
                        Df Sum Sq Mean Sq F value  Pr(>F)
data_stat_id$treatment  7   4867     695     340 1.6e-14 ***
Residuals              14     29       2
---
Signif. codes:  0 '***' 0.001 '**' 0.01 '*' 0.05 '.' 0.1 ' ' 1
[1] ""
[1] "############"
[1] ""
[1] "Dunnett-test results:"

  Dunnett's test for comparing several treatments with a control :
    95% family-wise confidence level

$Control
                   diff   lwr.ci    upr.ci    pval
pH = 2-Control -46.6667 -51.6075 -41.72579 < 2e-16 ***
pH = 3-Control -37.3333 -40.8271 -33.83961 < 2e-16 ***
pH = 4-Control  -4.0000  -7.4937  -0.50627  0.0221 *
pH = 5-Control  -1.3333  -4.8271   2.16039  0.7807
pH = 6-Control  -3.3333  -6.8271   0.16039  0.0644 .
pH = 7-Control -14.0000 -17.4937 -10.50627 1.6e-08 ***
pH = 8-Control -26.3333 -29.8271 -22.83961 < 2e-16 ***

---
Signif. codes:  0 '***' 0.001 '**' 0.01 '*' 0.05 '.' 0.1 ' ' 1

[1] ""
[1] "################################"
[1] "################################"
[1] ""
[1] "TD16"
```

Figure S36. pH − TD16

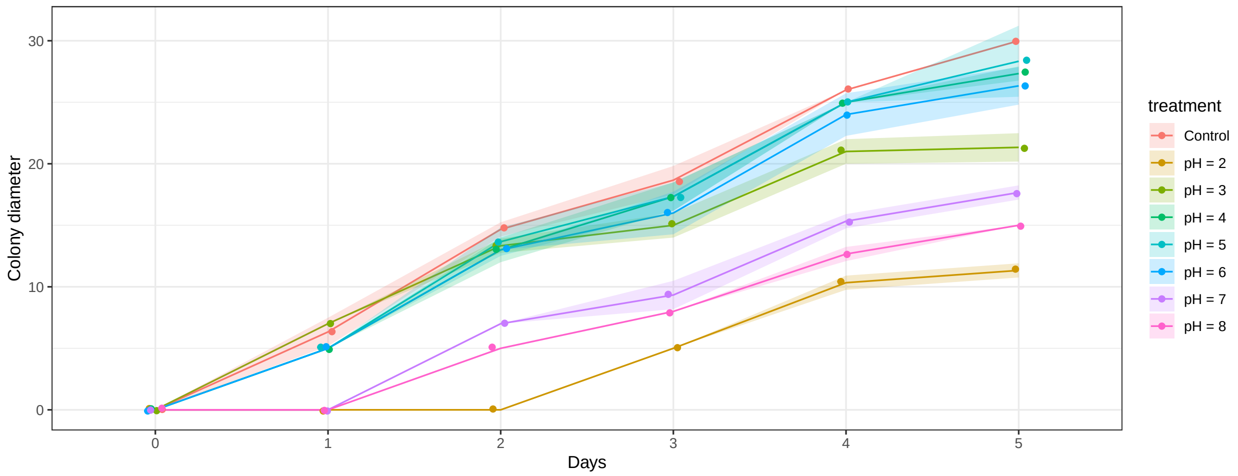


```
[1] "ANOVA results:"
                        Df Sum Sq Mean Sq F value  Pr(>F)    
data_stat_id$treatment  7   8199    1171     226 2.7e-13 ***
Residuals              14     73       5                    
---
Signif. codes:  0 '***' 0.001 '**' 0.01 '*' 0.05 '.' 0.1 ' ' 1
[1] ""
[1] "############"
[1] ""
[1] "Dunnett-test results:"

  Dunnett's test for comparing several treatments with a control :  
    95% family-wise confidence level

$Control
                    diff  lwr.ci    upr.ci    pval    
pH = 2-Control -15.6667 -23.533  -7.80014 0.00020 ***
pH = 3-Control -18.0000 -23.562 -12.43753 2.3e-06 ***
pH = 4-Control  -8.0000 -13.562  -2.43753 0.00418 ** 
pH = 5-Control  -6.3333 -11.896  -0.77086 0.02299 *  
pH = 6-Control -11.3333 -16.896  -5.77086 0.00018 ***
pH = 7-Control -46.3333 -51.896 -40.77086 < 2e-16 ***
pH = 8-Control -55.0000 -60.562 -49.43753 < 2e-16 ***

---
Signif. codes:  0 '***' 0.001 '**' 0.01 '*' 0.05 '.' 0.1 ' ' 1

[1] ""
[1] "################################"
[1] "################################"
[1] ""
[1] "TD22"
```

Figure S37. pH – TD22

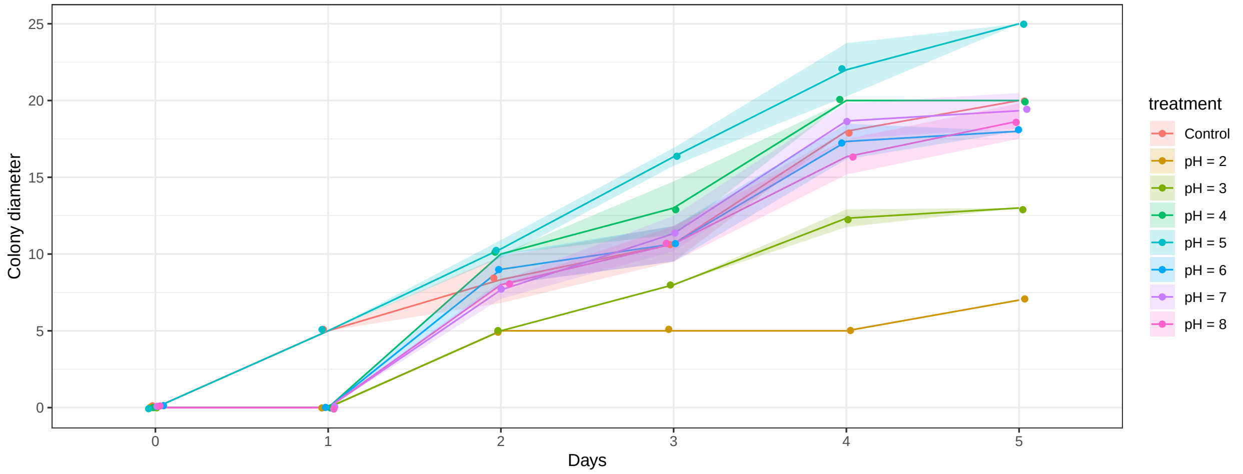


```
[1] "ANOVA results:"
                       Df Sum Sq Mean Sq F value  Pr(>F)    
data_stat_id$treatment  7   2707     387     129 1.3e-11 ***
Residuals              14     42       3                    
---
Signif. codes:  0 '***' 0.001 '**' 0.01 '*' 0.05 '.' 0.1 ' ' 1
[1] ""
[1] "############"
[1] ""
[1] "Dunnett-test results:"

  Dunnett's test for comparing several treatments with a control :  
    95% family-wise confidence level

$Control
                    diff   lwr.ci    upr.ci    pval    
pH = 2-Control   4.0000  -1.9805   9.98053  0.2758    
pH = 3-Control -23.6667 -27.8955 -19.43779 1.1e-12 ***
pH = 4-Control   1.0000  -3.2289   5.22888  0.9687    
pH = 5-Control  16.6667  12.4378  20.89554 1.2e-09 ***
pH = 6-Control  -7.0000 -11.2289  -2.77112  0.0014 ** 
pH = 7-Control  -5.0000  -9.2289  -0.77112  0.0178 *  
pH = 8-Control  -8.3333 -12.5622  -4.10446  0.0002 ***

---
Signif. codes:  0 '***' 0.001 '**' 0.01 '*' 0.05 '.' 0.1 ' ' 1

[1] ""
[1] "################################"
[1] "################################"
[1] ""
[1] "TD25"
```

Figure S38. pH − TD25

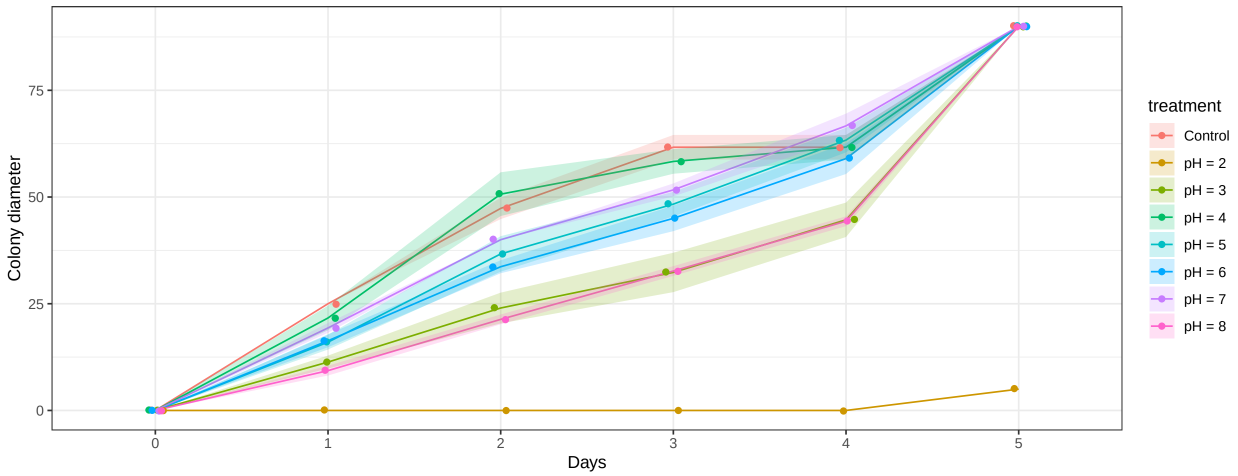


```
[1] "ANOVA results:"
                        Df Sum Sq Mean Sq F value  Pr(>F)
data_stat_id$treatment  7  74671   10667     254 1.2e-13 ***
Residuals              14    588      42
---
Signif. codes:  0 '***' 0.001 '**' 0.01 '*' 0.05 '.' 0.1 ' ' 1
[1] ""
[1] "############"
[1] ""
[1] "Dunnett-test results:"

  Dunnett's test for comparing several treatments with a control :
    95% family-wise confidence level

$Control
                     diff   lwr.ci   upr.ci    pval
pH = 2-Control -270.6667 -293.044 -248.290 < 2e-16 ***
pH = 3-Control  -83.3333  -99.156  -67.510 2.5e-13 ***
pH = 4-Control   -3.3333  -19.156   12.490 0.98251
pH = 5-Control  -31.3333  -47.156  -15.510 0.00024 ***
pH = 6-Control  -41.6667  -57.490  -25.844 8.2e-06 ***
pH = 7-Control  -18.0000  -33.823   -2.177 0.02330 *
pH = 8-Control  -88.0000 -103.823  -72.177 5.8e-15 ***

---
Signif. codes:  0 '***' 0.001 '**' 0.01 '*' 0.05 '.' 0.1 ' ' 1

[1] ""
[1] "################################"
[1] "################################"
[1] ""
[1] "TD4"
```

Figure S39. pH − TD4

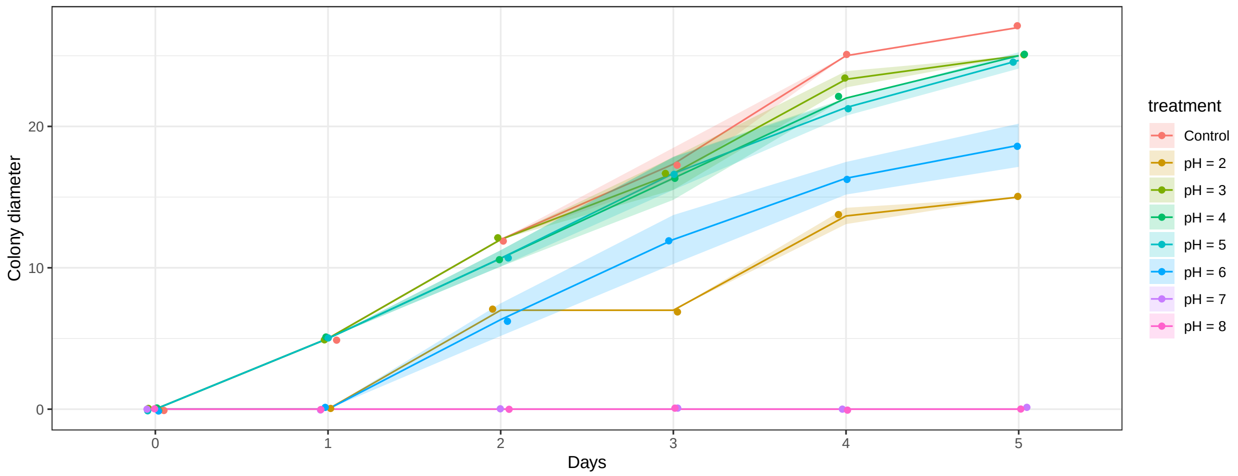


```
[1] "ANOVA results:"
                        Df Sum Sq Mean Sq F value Pr(>F)
data_stat_id$treatment  7  31844    4549    2450 <2e-16 ***
Residuals              14     26       2
---
Signif. codes:  0 '***' 0.001 '**' 0.01 '*' 0.05 '.' 0.1 ' ' 1
[1] ""
[1] "############"
[1] ""
[1] "Dunnett-test results:"

  Dunnett's test for comparing several treatments with a control :
    95% family-wise confidence level

$Control
                   diff   lwr.ci   upr.ci    pval
pH = 2-Control  41.6667  36.9612  46.3721 < 2e-16 ***
pH = 3-Control  -4.3333  -7.6606  -1.0061  0.0090 **
pH = 4-Control  -7.3333 -10.6606  -4.0061 9.9e-05 ***
pH = 5-Control  -8.0000 -11.3273  -4.6727 1.8e-05 ***
pH = 6-Control -33.0000 -36.3273 -29.6727 < 2e-16 ***
pH = 7-Control -86.3333 -89.6606 -83.0061 < 2e-16 ***
pH = 8-Control -86.3333 -89.6606 -83.0061 < 2e-16 ***

---
Signif. codes:  0 '***' 0.001 '**' 0.01 '*' 0.05 '.' 0.1 ' ' 1

[1] ""
[1] "################################"
[1] "################################"
[1] ""
[1] "TD5"
```

Figure S40. pH – TD5

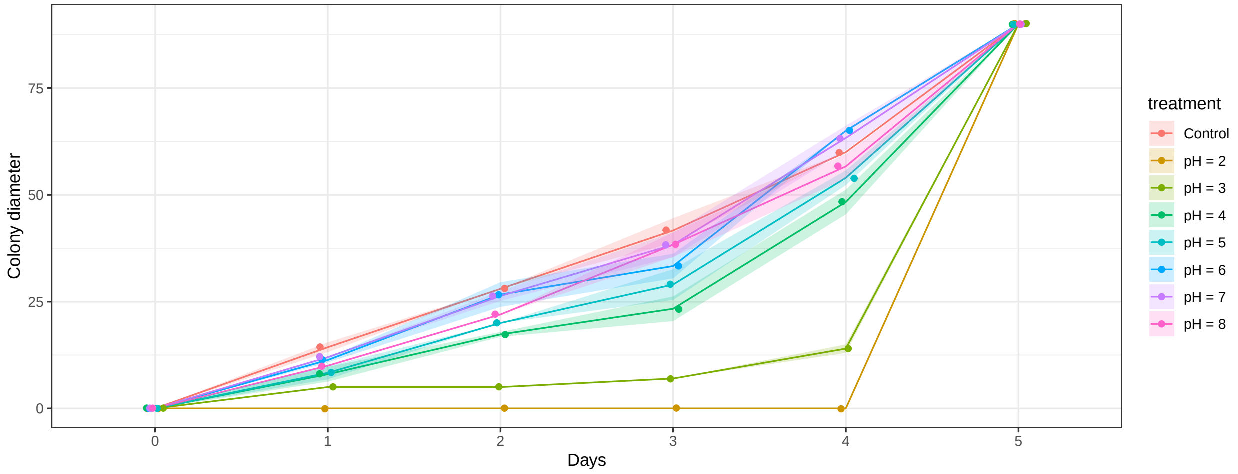


```
[1] "ANOVA results:"
                       Df Sum Sq Mean Sq F value  Pr(>F)    
data_stat_id$treatment  7  32596    4657     586 3.6e-16 ***
Residuals              14    111       8                    
---
Signif. codes:  0 '***' 0.001 '**' 0.01 '*' 0.05 '.' 0.1 ' ' 1
[1] ""
[1] "############"
[1] ""
[1] "Dunnett-test results:"

  Dunnett's test for comparing several treatments with a control :  
    95% family-wise confidence level

$Control
                    diff   lwr.ci      upr.ci    pval    
pH = 2-Control   36.0000   26.263   45.73706 5.4e-08 ***
pH = 3-Control -113.0000 -119.885 -106.11486 < 2e-16 ***
pH = 4-Control  -47.0000  -53.885  -40.11486 < 2e-16 ***
pH = 5-Control  -32.6667  -39.552  -25.78153 1.5e-10 ***
pH = 6-Control   -7.6667  -14.552   -0.78153  0.0263 *  
pH = 7-Control   -4.0000  -10.885    2.88514  0.4045    
pH = 8-Control  -17.0000  -23.885  -10.11486 1.1e-05 ***

---
Signif. codes:  0 '***' 0.001 '**' 0.01 '*' 0.05 '.' 0.1 ' ' 1

[1] ""
[1] "################################"
[1] "################################"
[1] ""
[1] "TD6"
```

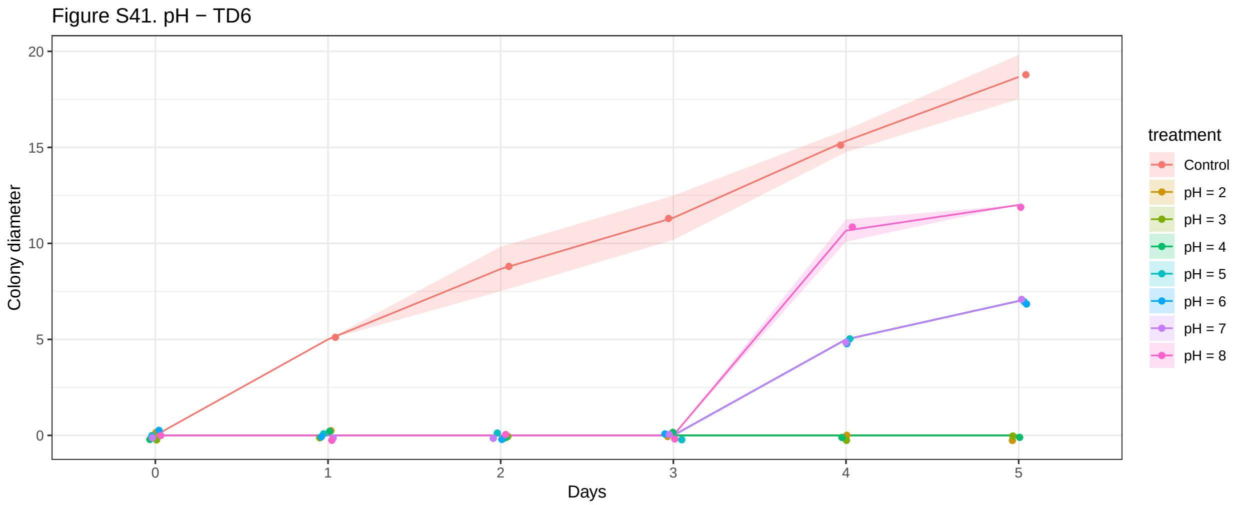


```
[1] "ANOVA results:"
                        Df Sum Sq Mean Sq F value Pr(>F)
data_stat_id$treatment  7   7616    1088    5712 <2e-16 ***
Residuals              14      3       0
---
Signif. codes:  0 '***' 0.001 '**' 0.01 '*' 0.05 '.' 0.1 ' ' 1
[1] ""
[1] "############"
[1] ""
[1] "Dunnett-test results:"

  Dunnett's test for comparing several treatments with a control :
    95% family-wise confidence level

$Control
                  diff  lwr.ci  upr.ci    pval
pH = 2-Control -59.000 -60.507 -57.493 <2e-16 ***
pH = 3-Control -59.000 -60.066 -57.934 <2e-16 ***
pH = 4-Control -59.000 -60.066 -57.934 <2e-16 ***
pH = 5-Control -47.000 -48.066 -45.934 <2e-16 ***
pH = 6-Control -47.000 -48.066 -45.934 <2e-16 ***
pH = 7-Control -47.000 -48.066 -45.934 <2e-16 ***
pH = 8-Control -36.333 -37.399 -35.268 <2e-16 ***

---
Signif. codes:  0 '***' 0.001 '**' 0.01 '*' 0.05 '.' 0.1 ' ' 1

[1] ""
[1] "################################"
[1] "################################"
[1] ""
[1] "TD8"
```

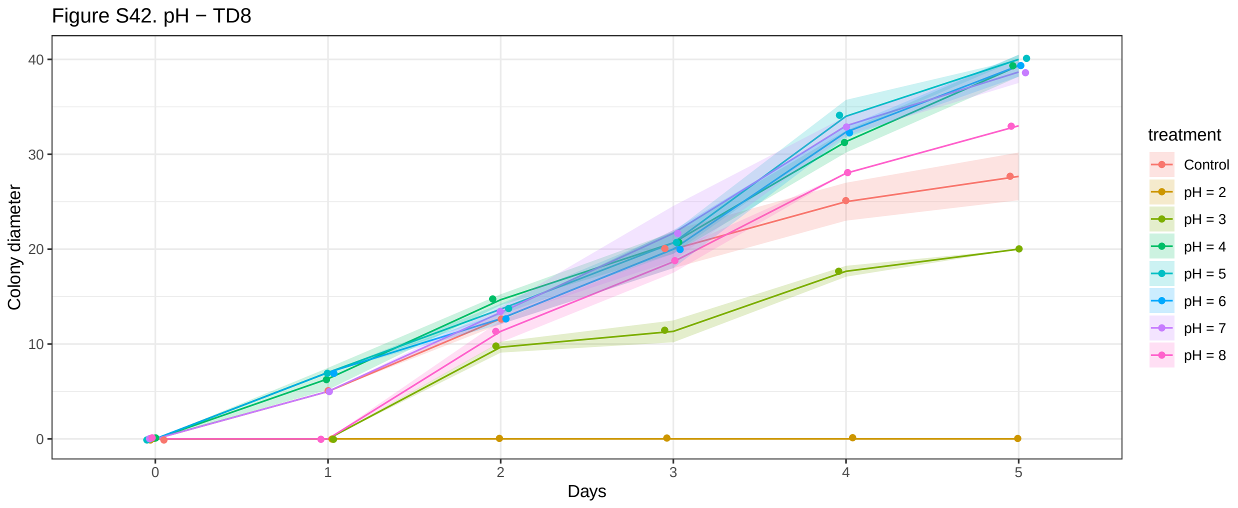


```
[1] "ANOVA results:"
                        Df Sum Sq Mean Sq F value  Pr(>F)    
data_stat_id$treatment  7  16859    2408     562 4.7e-16 ***
Residuals              14     60       4                    
---
Signif. codes:  0 '***' 0.001 '**' 0.01 '*' 0.05 '.' 0.1 ' ' 1
[1] ""
[1] "############"
[1] ""
[1] "Dunnett-test results:"

  Dunnett's test for comparing several treatments with a control :  
    95% family-wise confidence level

$Control
                 diff    lwr.ci    upr.ci    pval    
pH = 2-Control -90.33333 -97.4814 -83.1852 < 2e-16 ***
pH = 3-Control -31.66667 -36.7211 -26.6122 2.8e-15 ***
pH = 4-Control  22.00000  16.9455  27.0545 3.4e-11 ***
pH = 5-Control  25.00000  19.9455  30.0545 4.1e-10 ***
pH = 6-Control  21.00000  15.9455  26.0545 2.7e-09 ***
pH = 7-Control  21.33333  16.2789  26.3878 1.5e-11 ***
pH = 8-Control   0.66667  -4.3878   5.7211  0.9988    

---
Signif. codes:  0 '***' 0.001 '**' 0.01 '*' 0.05 '.' 0.1 ' ' 1

[1] ""
[1] "################################"
[1] "################################"
[1] ""
[1] "TD9"
```

Figure S43. pH − TD9

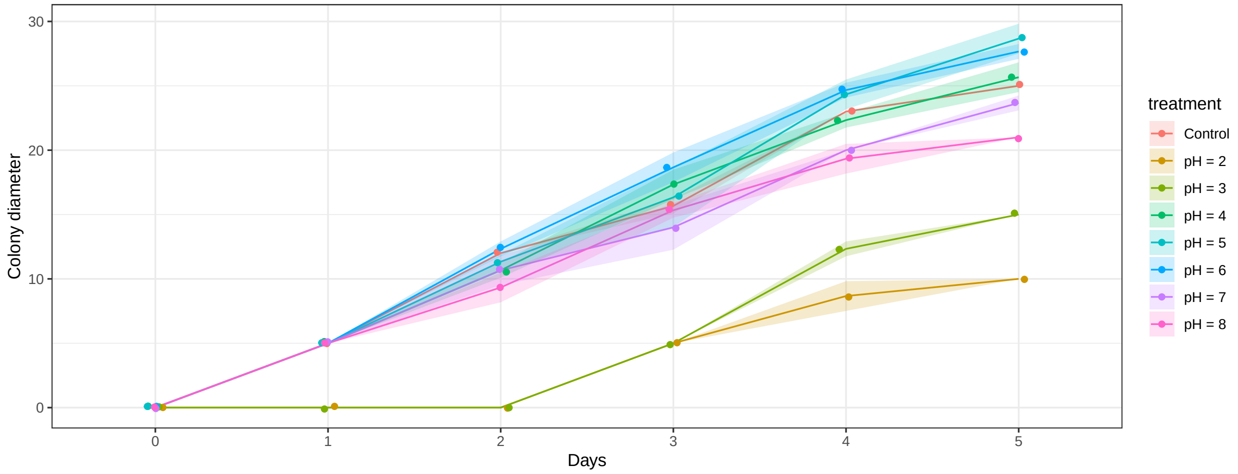


```
[1] "ANOVA results:"
                       Df Sum Sq Mean Sq F value  Pr(>F)    
data_stat_id$treatment  7   6548     935     265 8.7e-14 ***
Residuals              14     49       4                    
---
Signif. codes:  0 ’***’ 0.001 ’**’ 0.01 ’*’ 0.05 ’.’ 0.1 ’ ’ 1
[1] ""
[1] "############"
[1] ""
[1] "Dunnett-test results:"

  Dunnett’s test for comparing several treatments with a control :  
    95% family-wise confidence level

$Control
                    diff    lwr.ci   upr.ci    pval    
pH = 2-Control  -9.66667 -16.14832  -3.1850  0.0032 ** 
pH = 3-Control -48.33333 -52.91655 -43.7501 < 2e-16 ***
pH = 4-Control   0.33333  -4.24988   4.9166  1.0000    
pH = 5-Control   5.00000   0.41678   9.5832  0.0300 *  
pH = 6-Control   7.66667   3.08345  12.2499  0.0010 ** 
pH = 7-Control  -7.33333 -11.91655  -2.7501  0.0018 ** 
pH = 8-Control -10.66667 -15.24988  -6.0834 2.5e-05 ***

---
Signif. codes:  0 ’***’ 0.001 ’**’ 0.01 ’*’ 0.05 ’.’ 0.1 ’ ’ 1

[1] ""
[1] "################################"
[1] "################################"
[1] ""
```
